# Supplementary material for: Resilience of genetic diversity in forest trees over the Quaternary
Source: Nat Commun. 2024 Oct 14;15:8538. doi: 10.1038/s41467-024-52612-y (PMC11473659; doi:10.1038/s41467-024-52612-y)
Supplement: Supplementary file 1 — Supplementary Information [file 41467_2024_52612_MOESM1_ESM.pdf]

Supplementary Information

Resilience of genetic diversity in forest trees over the Quaternary

Pascal Milesi, Chedly Kastally, Benjamin Dauphin, Sandra Cervantes, Francesca Bagnoli, Katharina B. Budde, Stephen Cavers, Bruno Fady, Patricia Faivre-Rampant, Santiago C González-Martínez, Delphine Grivet, Felix Gugerli, Véronique Jorge, Isabelle Lesur Kupin, Dario I. Ojeda, Sanna Olsson, Lars Opgenoorth, Sara Pinosio, Christophe Plomion, Christian Rellstab, Odile Rogier, Simone Scalabrin, Ivan Scotti, Giovanni G Vendramin, Marjana Westergren, Martin Lascoux, Tanja Pyhäjärvi on behalf of the GenTree Consortium.

Correspondence to: pascal.milesi@scilifelab.uu.se martin.lascoux@ebc.uu.se and tanja.pyhajarvi@helsinki.fi

This PDF file includes:

Supplementary Materials and Methods
Figures S1 to S33
Tables S1 to S10

Other Supplementary Materials for this manuscript include the following:

Supplementary Data 1: Sampling location IDs, species, coordinates (WGS84) and number of individuals in vcf v.5.3.1.

Supplementary Data 2: Coordinates, expected heterozygosity at all, silent, 4-fold and 0-fold degenerate sites, and population-specific FST per population and species.

Supplementary Data 3: Genetic distance and geographic distance (as pairwise FST) among all pairs of populations.

Supplementary Data 4: AIC, delta AIC and wi computed for each species, sampling design and model.

Supplementary Data 5: Confidence intervals (95%) of the parameter obtained with the panmictic models (2-epoch and 3-epoch, with one and two demographic changes, respectively) across all species and the two sampling designs (AllSamples = all samples included, OnePerPop = one haplotype per location included). The median and the 2.5 and 97.5 percentiles are provided for each parameter. NANC = population effective size of the ancestral population; NCUR = population effective size of the current population; TLATE = time in generations of the demographic event in the 2-epoch model and the most ancient demographic event in the 3-epoch model; TEARLY = time in generations of the most recent demographic event in the 3-epoch model; TLATE_year = same as TLATE but

expressed in years; TEARLY_year = same as TLATE but expressed in year; Ratio NCUR/NANC = ratio of NCUR and NANC.

Supplementary Data 6: Single-point estimates for several parameters obtained from simulations under seven models (SNM, 2-epoch, 3-epoch, div-e1-iso, div-e2-iso, div-e1-mig, div-e2-mig) across four sampling designs (AllSamples, OnePerPop, PairPop, SinglePop) with fastsimcoal2. These estimates were taken from the best run out of 100 independent runs (see Materials and Methods).

Supplementary Data 7: All results obtained with Stairway Plot 2 concatenated into a single file with columns ‘species’ and ‘population’ specific to each individual analysis. Species-wide analyses were run using two sampling designs, AllSamples and OnePerPop, and are identified as such in the ‘population’ column. For population-wide analyses the population ID is given in the ‘population’ column.

Supplementary Data 8: Gene Ontology (GO) and Kyoto Encyclopedia of Genes and Genomes (KEGG) terms for selected genes

Supplementary Data 9: The set of 811 orthogroups that included at least one gene for at least six of the seven tree species.

Supplementary Data 10: Other ‘orthogroups’ including genes that were found only in a reduced set of species (e.g. 59 additional genes across the three conifer species).

GenTree consortium author list

Audrey Albet[1], Ricardo Alía[2], Paraskevi Alizoti[3], Olivier Ambrosio[4], Filippos A. Aravanopoulos[2], Francisco Auñón[2], Camilla Avanzi[5], Evangelia Avramidou[3], Marko Bajc[6], Eduardo Ballesteros[2], Evangelos Barbas[3], Cristina C. Bastias[7], Catherine Bastien[8], Giorgia Beffa[9,10], Raquel Benavides[11], Vanina Benoit[12], Frédéric Bernier[1], Henri Bignalet[1], Guillaume Bodineau[13], Damien Bouic[1], Sabine Brodbeck[9], William Brunetto[4], Jurata Buchovska[14], Corinne Buret[12], Melanie Buy[15], Ana M. Cabanillas-Saldaña[16], Bárbara Carvalho[11], Nicolas Cheval[1], José M. Climent[2], Marianne Correard[17], Eva Cremer[18], Darius Danusevičius[14], Nicolas De Girardi[9], Fernando Del Caño[2], Jean-Luc Denou[1], Bernard Dokhelar[1], Remi Dourthe[1], Alexis Ducousso[20], Anna-Maria Farsakoglou[3], Patrick Fonti[9], Ioannis Ganopoulos[21], José M. García del Barrio[2], Olivier Gilg[17], René Graf[9], Alan Gray[22], Christoph Hartleitner[23], Katrin Heer[24], Enja Hollenbach[25], Agathe Hurel[19], Bernard Issenhuth[1], Florence Jean[4], Arnaud Jouineau[3], Jan-Philipp Kappner[25], Katri Kärkkäinen[26], Robert Kesälahti[27], Florian Knutzen[18], Sonja T. Kujala[26], Timo A. Kumpula[27], Mariaceleste Labriola[28], Celine Lalanne[19], Johannes Lambertz[25], Gregoire Le-Provost[19], Vincent Lejeune[13], Joseph Levillain[29], Mirko Liesebach[30], David López-Quiroga[11], Ermioni Malliarou[3], Jérémy Marchon[9], Nicolas Mariotte[4], Elisabet Martínez-Sancho[9], Antonio Mas[11], Silvia Matesanz[31], Benjamin Meier[9], Helge Meischner[25], Célia Michotey[15], Sandro Morganti[9], Tor Myking[20], Daniel Nievergelt[9], Anne Eskild Nilsen[20], Eduardo Notivol[32], Geir Ostreng[21], Birte Pakull[31], Annika Perry[23], Andrea Piotti[6], Nicolas Poinot[5], Mehdi Pringarbe[18], Luc Puzos[5], Annie Raffin[5], José A. Ramírez-Valiente[3], Oliver Reutimann[9,33], Sebastian Richter[25], Juan J. Robledo-Arnuncio[2], Sergio San Segundo[2], Outi Savolainen[27], Volker Schneck[34], Silvio Schueler[35], Vladimir Semerikov[36], Lenka Slámová[9,37], Jørn Henrik Sønstebø[20], Ilaria Spanu[5], Jean Thevenet[17], Mari Mette Tollefsrud[20], Norbert Turion[17], Fernando Valladares[11], Marc Villar[12], Georg von Arx[9], Johan Westin[38]

Affiliations:
[1]INRAE, UEFP, 33610 Cestas, France
[2]Institute of Forest Sciences (ICIFOR-INIA), CSIC, 28040 Madrid, Spain
[3]Aristotle University of Thessaloniki, School of Forestry and Natural Environment, Laboratory of Forest Genetics and Tree Improvement, 54124 Thessaloniki, Greece
[4]INRAE, URFM, 84914 Avignon, France
[5]Institute of Biosciences and Bioresources, National Reasearch Council of Italy (IBBR-CNR), 50019 Sesto Fiorentino, Italy
[6]Slovenian Forestry Institute, 1000 Ljubljana, Slovenia
[7]CEFE, CNRS, UMR 5175, 34090 Montpellier, France
[8]INRAE, ECODIV, 45075 Orléans, France
[9]Swiss Federal Research Institute WSL, 8903 Birmensdorf, Switzerland
[10]Institute of Plant Sciences, University of Bern, 3013 Bern, Switzerland
[11]Department of Biogeography and Global Change, Museo Nacional de Ciencias Naturales, CSIC, 28006 Madrid, Spain
[12]INRAE, ONF, BioForA, 45075 Orléans, France
[13]INRAE, GBFOR, 45075 Orléans, France
[14]Vytautas Magnus University, 53361 Akademija, Lithuania
[15]INRAE, URGI, 78026 Versailles, France
[16]Departamento de Agricultura, Ganadería y Medio Ambiente, Gobierno de Aragón, 50071 Zaragoza, Spain

[17]INRAE, UEFM, 84914 Avignon, France
[18]Bavarian Institute for Forest Genetics, 83317 Teisendorf, Germany
[19]University of Bordeaux, INRAE, BIOGECO, 33770 Cestas, France
[20]Division of Forestry and Forest Resources, Norwegian Institute of Bioeconomy Research (NIBIO), 1431 Ås, Norway
[21]Institute of Plant Breeding and Genetic Resources, Hellenic Agricultural Organization DEMETER, 57001 Thermi, Greece
[22]UK Centre for Ecology & Hydrology (UKCEH), EH26 0QB Bush Estate, United Kingdom
[23]LIECO GmbH & Co KG, 8775 Kalwang, Austria
[24]Forest Genetics, Albert-Ludwigs Universität Freiburg, 79098 Freiburg, Germany
[25]Faculty of Biology, Plant Ecology and Geobotany, Philipps University Marburg, 35043 Marburg, Germany
[26]Natural Resources Institute Finland, University of Oulu, 90014 Oulu, Finland
[27]University of Oulu, 90014 Oulu, Finland
[28]Institute of Biosciences and Bioresources, National Reasearch Council of Italy (IBBR-CNR), 50019 Sesto Fiorentino, Italy
[29]Université de Lorraine, AgroParisTech, INRAE, SILVA, 54000 Nancy, France
[30]Thünen Institute of Forest Genetics, 22927 Grosshansdorf, Germany
[31]Área de Biodiversidad y Conservación, Universidad Rey Juan Carlos, 28933 Móstoles, Spain
[32]Centro de Investigación y Tecnología Agroalimentaria de Aragón, Dpto. de Sistemas Agrarios, Forestales y Medio Ambiente (CITA), 50059 Zaragoza, Spain
[33]Department of Environmental Systems Sciences, ETH Zurich, 8092 Zurich, Switzerland
[34]Thünen Institute of Forest Genetics, 15377 Waldsieversdorf, Germany
[35]Austrian Research Centre for Forests (BFW), 1131 Wien, Austria
[36]Institute of Plant and Animal Ecology, Ural branch of RAS, 620144 Ekaterinburg, Russia
[37]Institute for Environmental Sciences, University of Geneva, 1205 Geneva, Switzerland
[38]Skogforsk, 91821 Sävar, Sweden

GenTree consortium whole author list*
*Family name alphabetical order

Audrey Albet
Ricardo Alía
Paraskevi Alizoti
Olivier Ambrosio
Filippos A. Aravanopoulos
Francisco Auñón
Camilla Avanzi
Evangelia Avramidou
Francesca Bagnoli
Marko Bajc
Eduardo Ballesteros
Evangelos Barbas
Cristina C. Bastias
Catherine Bastien
Giorgia Beffa,
Raquel Benavides
Vanina Benoit
Frédéric Bernier
Henri Bignalet
Guillaume Bodineau
Damien Bouic
Sabine Brodbeck
William Brunetto
Jurata Buchovska
Katharina B. Budde
Corinne Buret
Melanie Buy
Ana M. Cabanillas-Saldaña
Bárbara Carvalho
Stephen Cavers
Sandra Cervantes
Nicolas Cheval
José M. Climent
Marianne Correard
Eva Cremer
Darius Danusevičius
Benjamin Dauphin
Nicolas De Girardi
Fernando Del Caño
Jean-Luc Denou
Bernard Dokhelar
Remi Dourthe
Alexis Ducousso
Anne Eskild Nilsen
Bruno Fady
Patricia Faivre-Rampant
Anna-Maria Farsakoglou
Patrick Fonti
Ioannis Ganopoulos
José M. García
Olivier Gilg
Santiago C. González-Martínez
René Graf
Alan Gray
Delphine Grivet
Felix Gugerli
Christoph Hartleitner
Katrin Heer
Jørn Henrik Sønstebø
Enja Hollenbach
Agathe Hurel
Bernard Issenhuth
Florence Jean
Véronique Jorge
Arnaud Jouineau
Jan-Philipp Kappner
Katri Kärkkäinen
Chedly Kastally
Robert Kesälahti
Florian Knutzen
Sonja T. Kujala
Timo A. Kumpula
Mariaceleste Labriola
Celine Lalanne
Johannes Lambertz
Martin Lascoux
Gregoire Le-Provost
Vincent Lejeune
Isabelle Lesur Kupin
Joseph Levillain
Mirko Liesebach
David López-Quiroga
Ermioni Malliarou
Jérémy Marchon
Nicolas Mariotte
Elisabet Martínez-Sancho
Antonio Mas
Silvia Matesanz
Benjamin Meier
Helge Meischner
Mari Mette Tollefsrud
Célia Michotey
Pascal Milesi
Sandro Morganti
Tor Myking
Daniel Nievergelt
Eduardo Notivol
Dario I. Ojeda
Sanna Olsson
Lars Opgenoorth
Geir Ostreng
Birte Pakull
Annika Perry
Sara Pinosio
Andrea Piotti
Christophe Plomion
Nicolas Poinot
Mehdi Pringarbe
Luc Puzos
Tanja Pyhäjärvi
Annie Raffin
José A. Ramírez-Valiente
Christian Rellstab
Oliver Reutimann,
Sebastian Richter
Juan J. Robledo-Arnuncio
Odile Rogier
Sergio San Segundo
Outi Savolainen
Simone Scalabrin
Volker Schneck
Silvio Schueler
Ivan Scotti
Vladimir Semerikov
Lenka Slámová,
Ilaria Spanu
Jean Thevenet
Norbert Turion
Fernando Valladares
Giovanni G. Vendramin
Marc Villar
Georg von Arx
Marjana Westergren
Johan Westin

Supplementary Materials and Methods

Dataset descriptions

As different types of analyses require SNP sets curated and filtered based on their specific needs, we provide four versions of SNP sets as vcf files available at https://doi.org/10.57745/DV2X0M. Note that since several species were observed to have experienced various levels and extents of hybridization, some admixed populations were included/excluded based on the purpose of a particular analysis. For example, in the analyses of population structure, we aimed to identify the potential admixed individuals and populations. In contrast, admixed individuals and populations can have a disproportionate effect on the measures of diversity and on the site frequency spectrum and were thus excluded from the corresponding analyses.

v.5.3 Known other species and clear hybrids removed, samples and SNPs with poor coverage or other low quality removed, organelle contigs removed (described in SNP filtering), *P. nigra* clones and cultivars removed, vcf format.
v.5.3.1 Master dataset derived from v.5.3, without samples with incorrect taxon assignment as indicated by genetic analysis for *F. sylvatica* and *Q. petraea*, identical to v.5.3 for the other five species, vcf format.
v.5.3.2 Derived from v.5.3.1, excludes *P. abies* populations RU_PA_19 and RU_PA_20, vcf format.
v.6.3.1 Only includes four-fold degenerate sites, intron and intergenic sites, SNPs in high LD (1 kb windows, $r > 0.5$, PLINK v.1.90b4.9[1,2], were excluded, derived from v.5.3.1
v.6.3.2 Derived from v.6.3.1, excludes singletons, ped- and map- format.

Clone and incorrect taxon identification and filtering

We identified *Populus* x *canadensis* genotypes among *P. nigra* samples based on an excess of heterozygous positions (> 5% of calls). We confirmed these by comparing their genotype at SSR markers with the INRAE database of common *Populus* x *canadensis* cultivars (https://urgi.versailles.inrae.fr/faidare/studies/dXJuOlVSR0kvc3R1ZHkvNjU%3D). We detected introgression from the ornamental *P. nigra* 'Italica' cultivar by using this cultivar as a control in the targeted capture experiment and performing ancestry analysis using ADMIXTURE software[3] on the whole SNP dataset. We discarded samples with a percentage of co-ancestry with 'Italica' > 85%, except for one Spanish genotype (ES_PO_01_02) that is identical to 'Italica' and represents the genotype. We identified clones through an identity by state (IBS) calculation on the whole SNP dataset using plink software (v.1.9).

For *Q. petraea*, we removed samples with an incorrect taxon identification by comparing cluster membership in a preliminary admixture analysis (see below) with the leaf hair density and type of herbarium proofs[4]. We identified as *Q. robur* 38 individuals forming their own cluster at K=2 and removed them for further analysis. These included seven individuals from population DE_QP_17, two from GB_QP_12, two from PL_QP_19, five from SE_QP_16, and the complete population SE_QP_15 (22 individuals).

We removed *F. sylvatica* population GR_FS_10 from the dataset because preliminary analyses of genotypic data by PCA showed it to be entirely different from the remaining populations. This stand is possibly composed of hybrids between *F. sylvatica* and *F. orientalis*.

For *P. abies*, *Picea obovata* served as an outgroup for interpreting admixture results. We excluded these samples from subsequent analyses, as well as samples from populations RU_PA_19 and RU_PA_20, which showed excessive admixture with *P. obovata*, and two *P. omorika* samples.

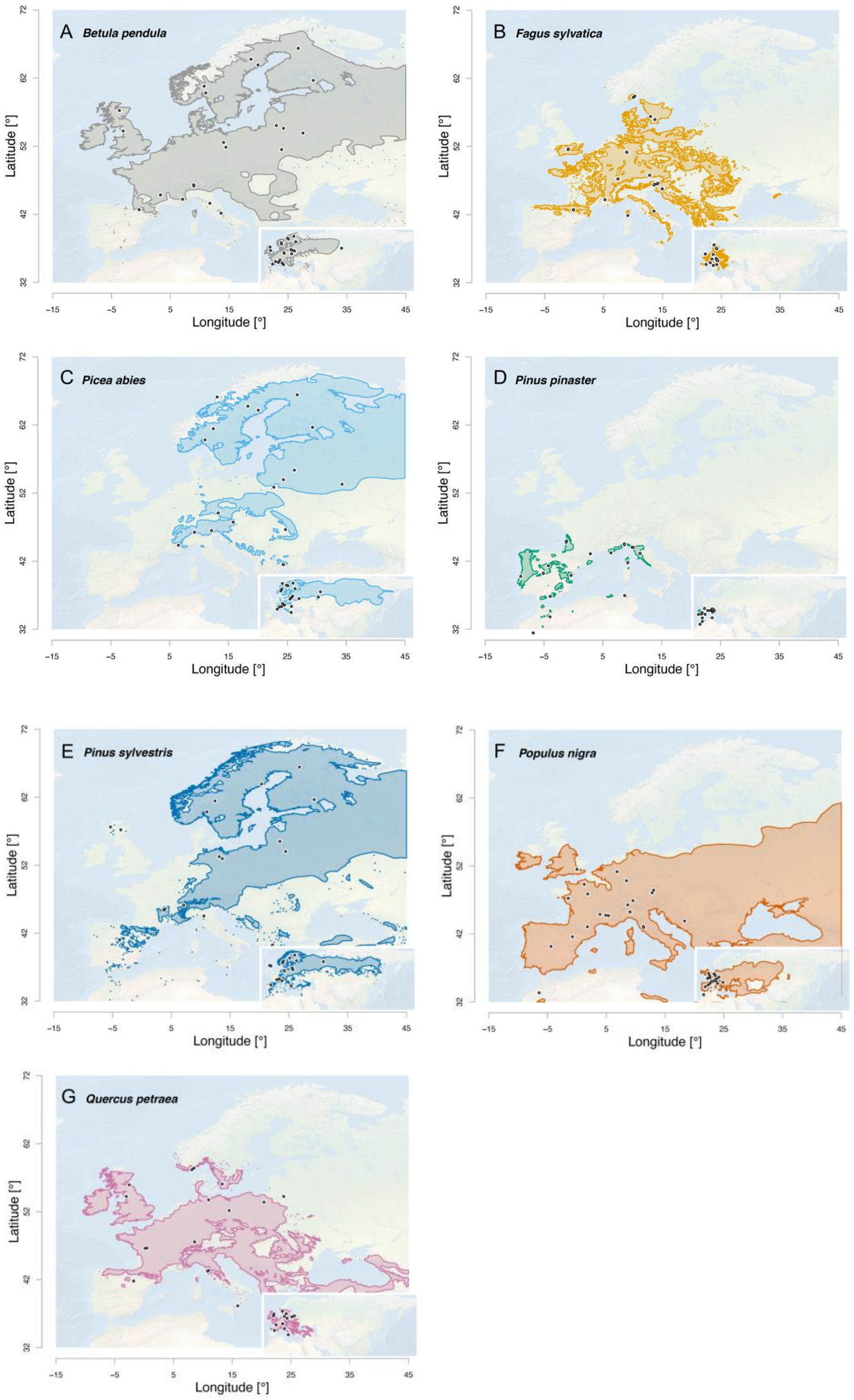

Figure S1. Sampling design. Species range[5] and sampling locations (black dots) for the seven species analyzed in this study.

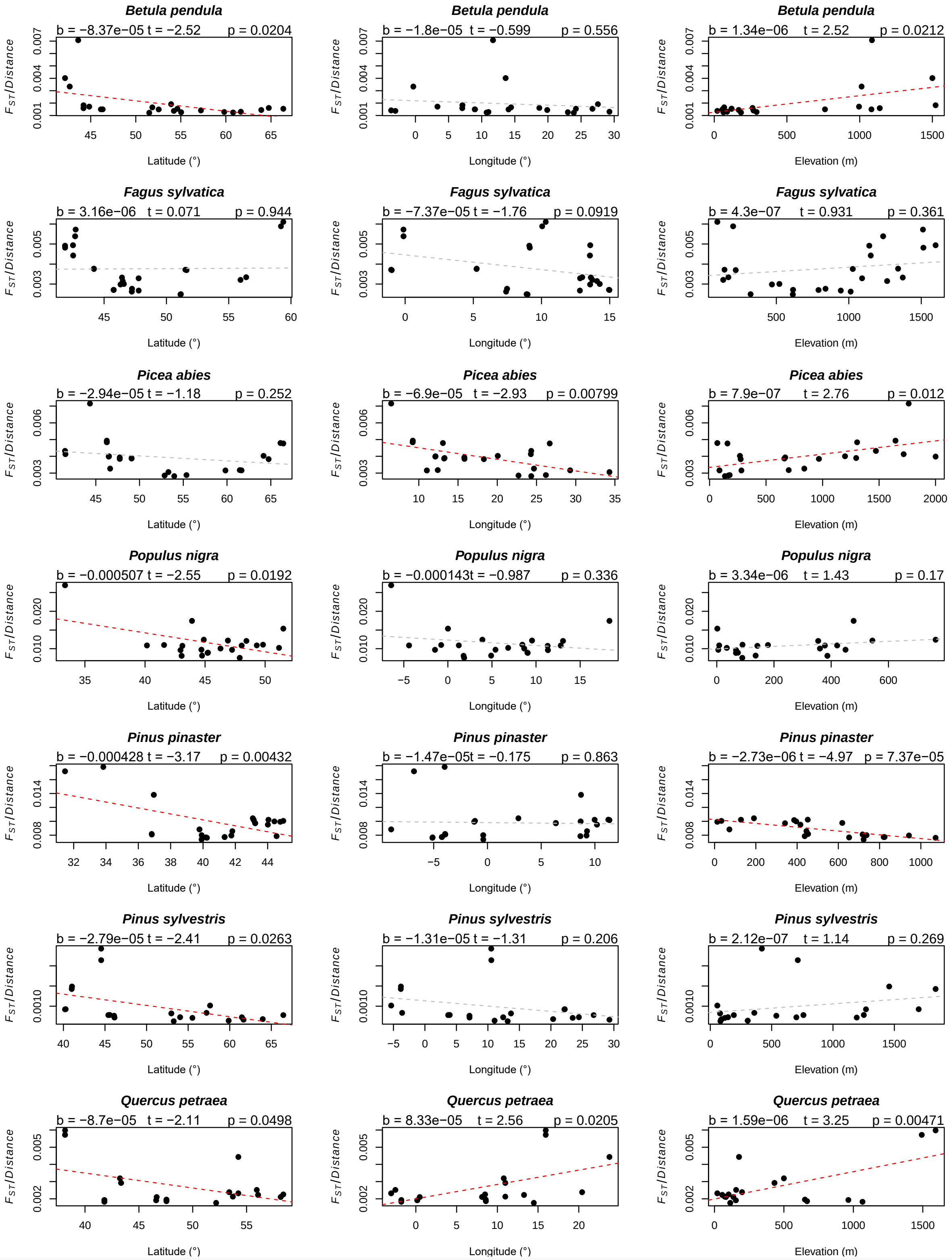

Figure S2. Variation in population-specific F_{ST} across space. Population-specific F_{ST} (average F_{ST} divided by average log distance) were regressed over latitude, longitude or elevation. The value of the slope of the linear regressions (b) and the *t*- and *P*-values are presented above the plot. Where $P < 0.05$ the regression line is shown in red.

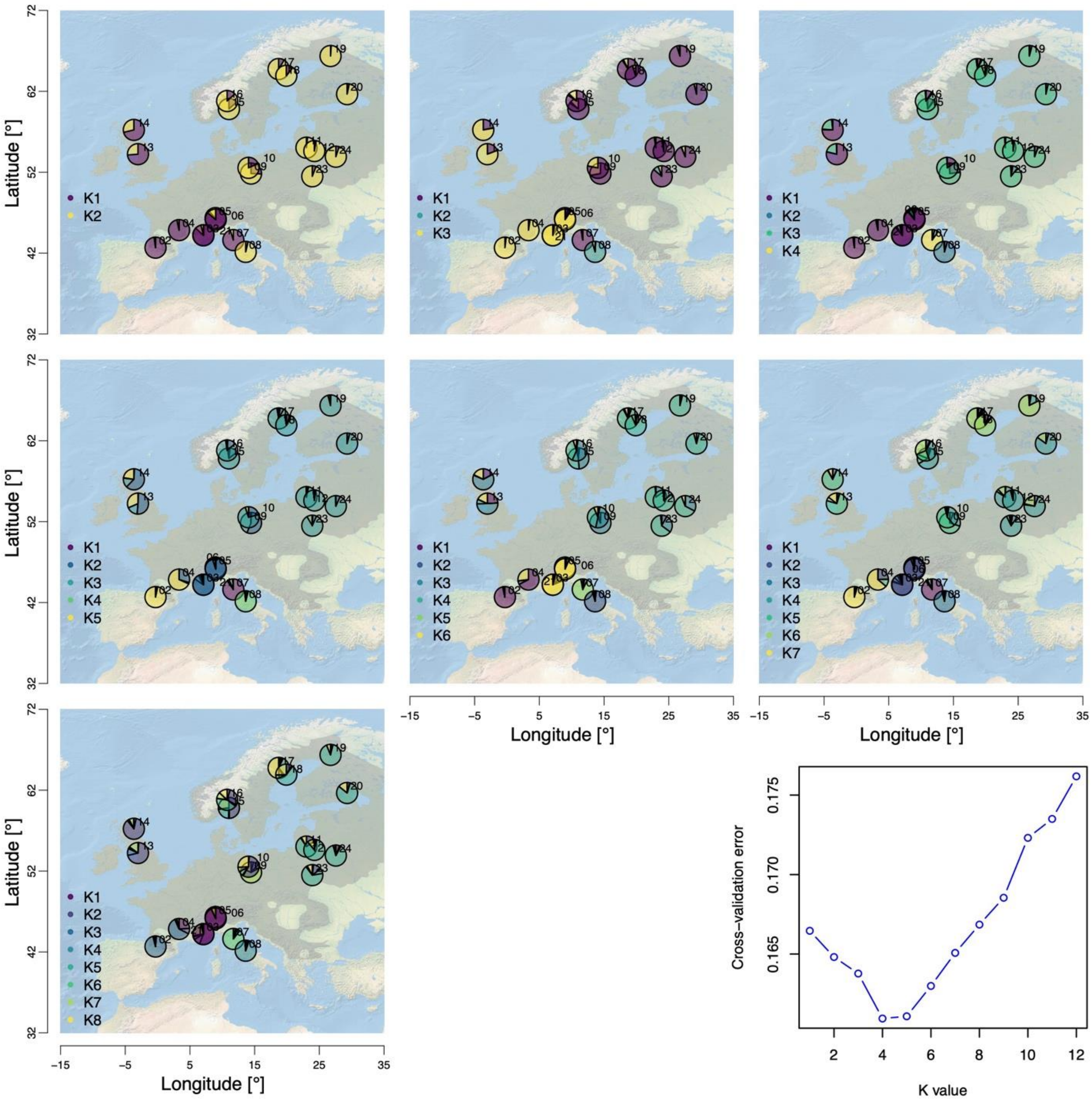

Figure S3. Admixture analysis among populations of *Betula pendula* based on the targeted capture SNP dataset. Geographic distribution[5] of the level of admixture for each population, with colors in pie charts reflecting average assignment probabilities to the respective genetic group (K = 2–8). Population codes are explained in Supplementary Data 1. The variation in cross-validation error across K values is represented in the bottom right panel, with the lowest value being the optimal number of genetic groups. Source data are provided as a Source Data file-1.

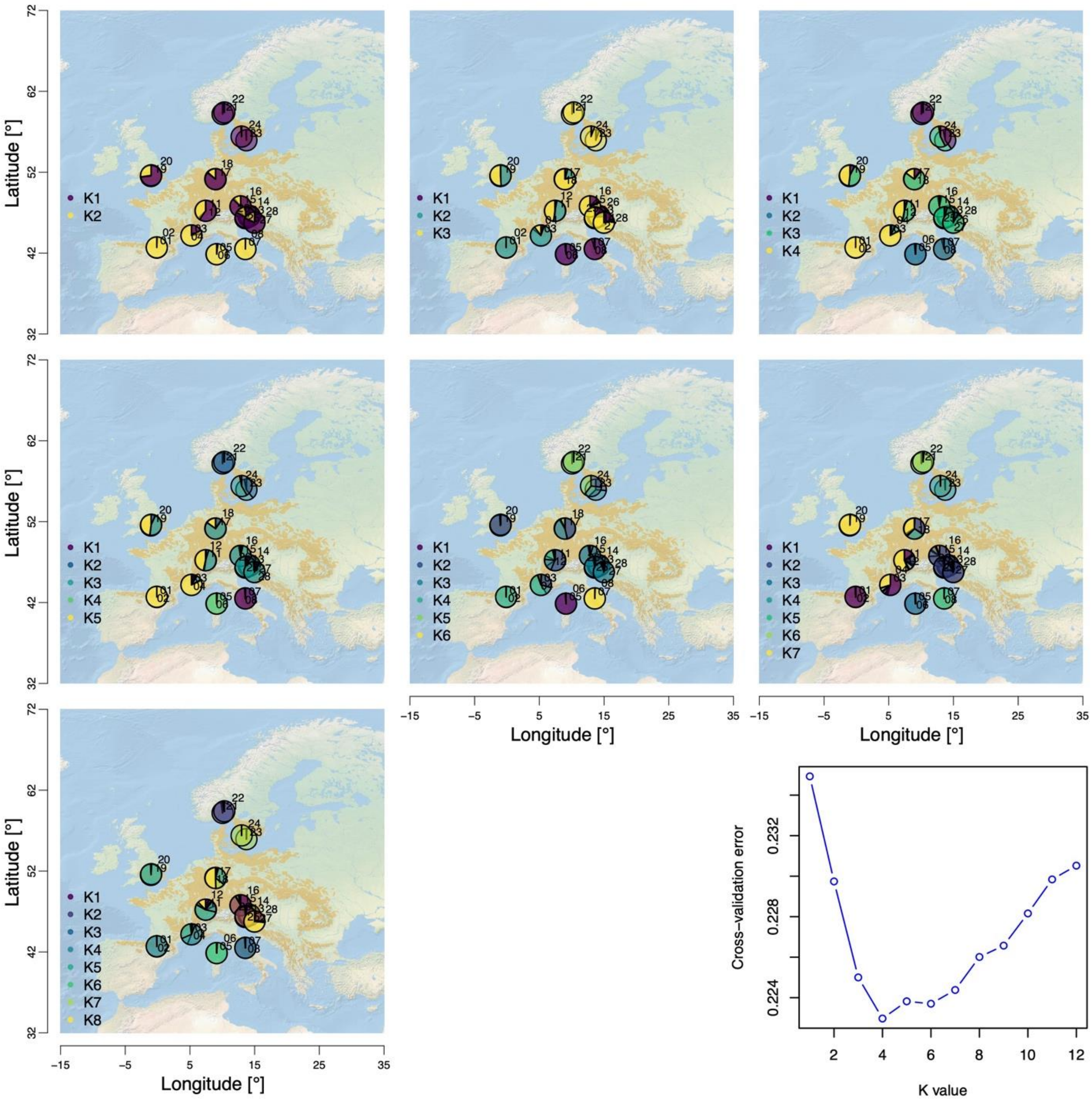

Figure S4. Admixture analysis among populations of *Fagus sylvatica* based on the targeted capture SNP dataset. Geographic distribution[5] of the level of admixture for each population, with colors in pie charts reflecting average assignment probabilities to the respective genetic group (*K* = 2–8). Population codes are explained in Supplementary Data 1. The variation in cross-validation error across *K* values is represented in the bottom right panel, with the lowest value being the optimal number of genetic groups. Source data are provided as a Source Data file-1.

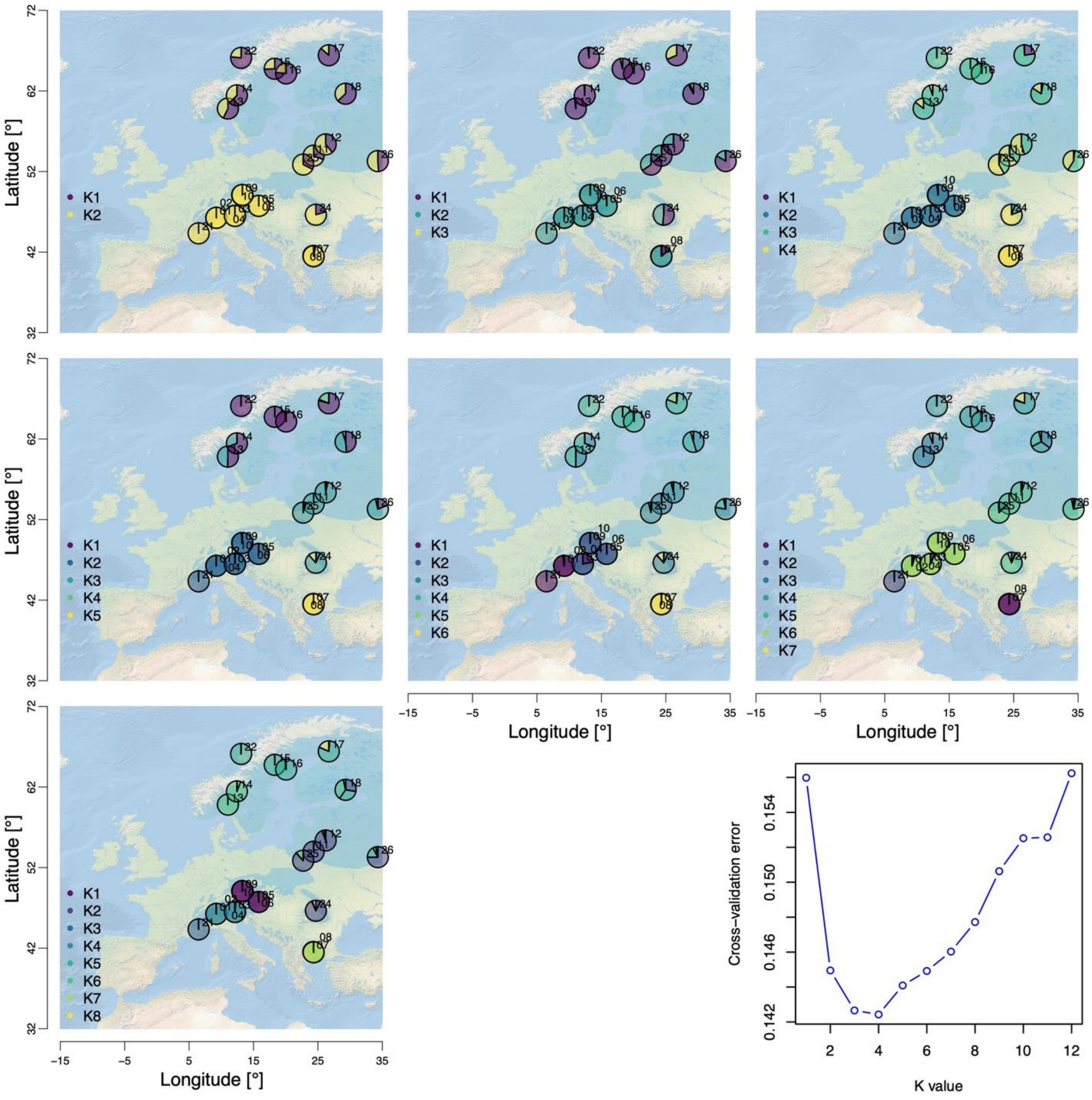

Figure S5. Admixture analysis among populations of *Picea abies* based on the targeted capture SNP dataset. Geographic distribution[5] of the level of admixture for each population, with colors in pie charts reflecting average assignment probabilities to the respective genetic group (K = 2–8). Population codes are explained in Supplementary Data 1. The variation in cross-validation error across K values is represented in the bottom right panel, with the lowest value being the optimal number of genetic groups. Source data are provided as a Source Data file-1.

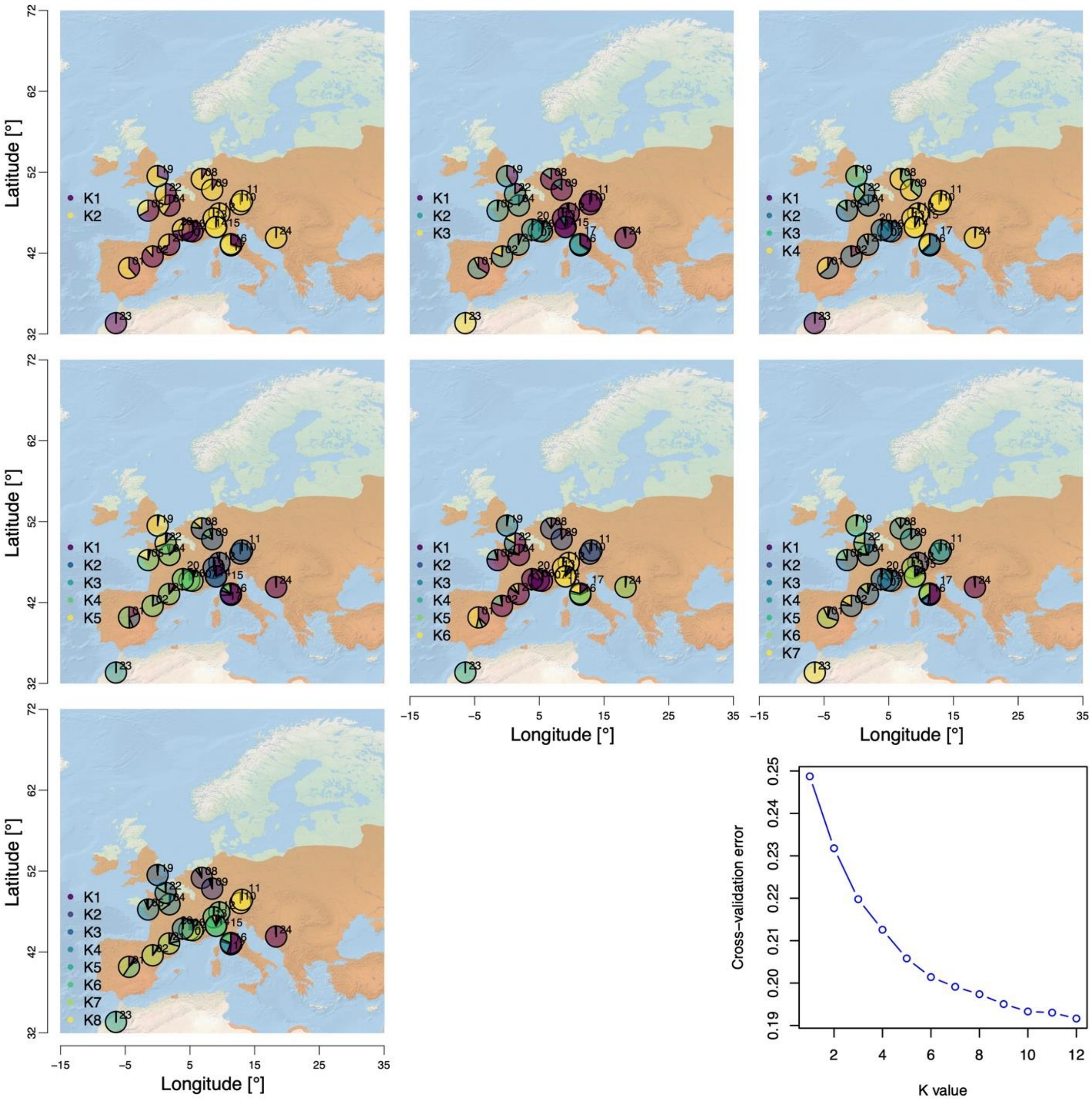

Figure S6. Admixture analysis among populations of *Populus nigra* based on the targeted capture SNP dataset. Geographic distribution[5] of the level of admixture for each population, with colors in pie charts reflecting average assignment probabilities to the respective genetic group (K = 2–8). Population codes are explained in Supplementary Data 1. The variation in cross-validation error across K values is represented in the bottom right panel, with the lowest value being the optimal number of genetic groups. Source data are provided as a Source Data file-1.

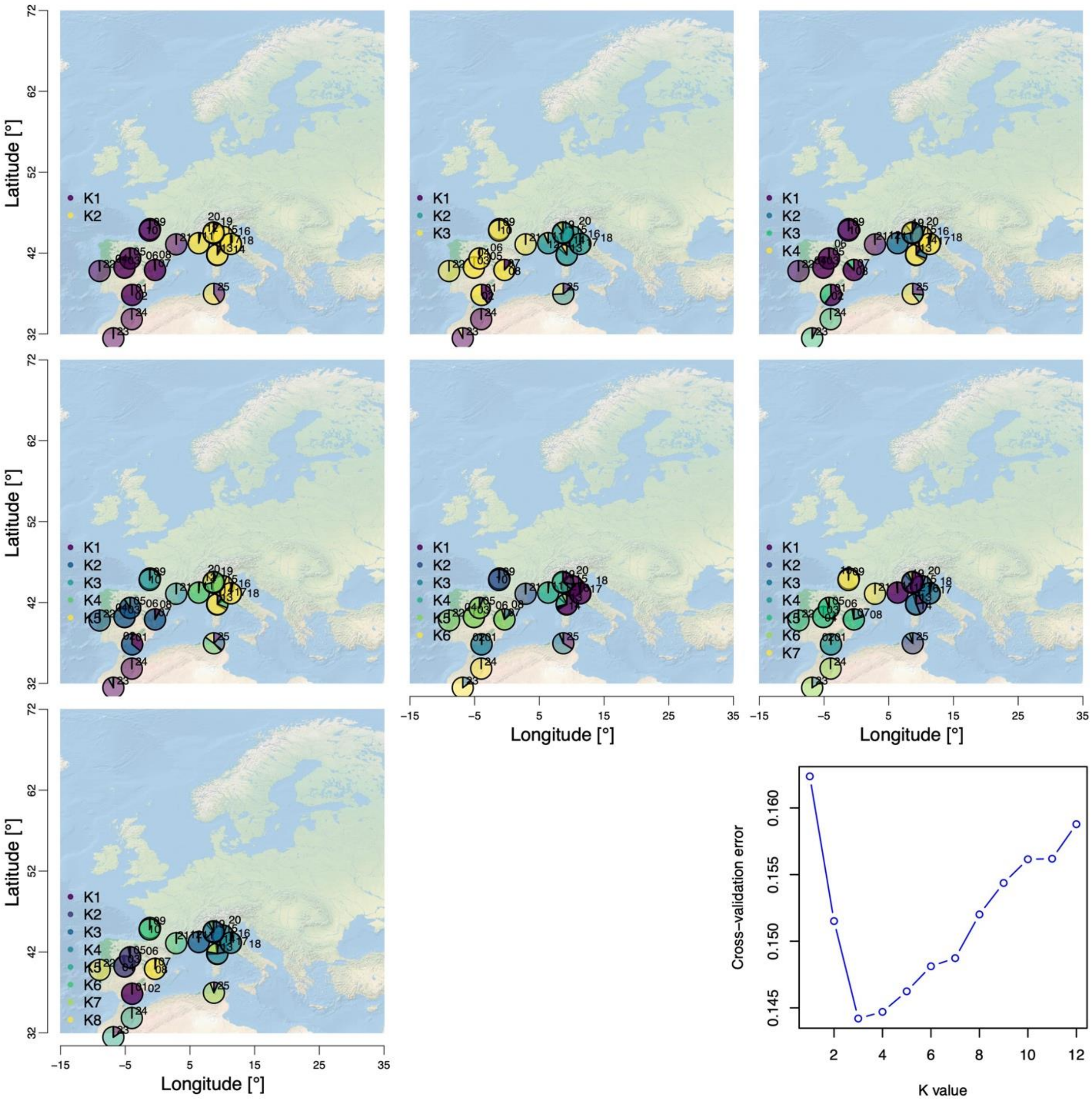

Figure S7. Admixture analysis among populations of *Pinus pinaster* based on the targeted capture SNP dataset. Geographic distribution[5] of the level of admixture for each population, with colors in pie charts reflecting average assignment probabilities to the respective genetic group (K = 2–8). Population codes are explained in Supplementary Data 1. The variation in cross-validation error across K values is represented in the bottom right panel, with the lowest value being the optimal number of genetic groups. Source data are provided as a Source Data file-1.

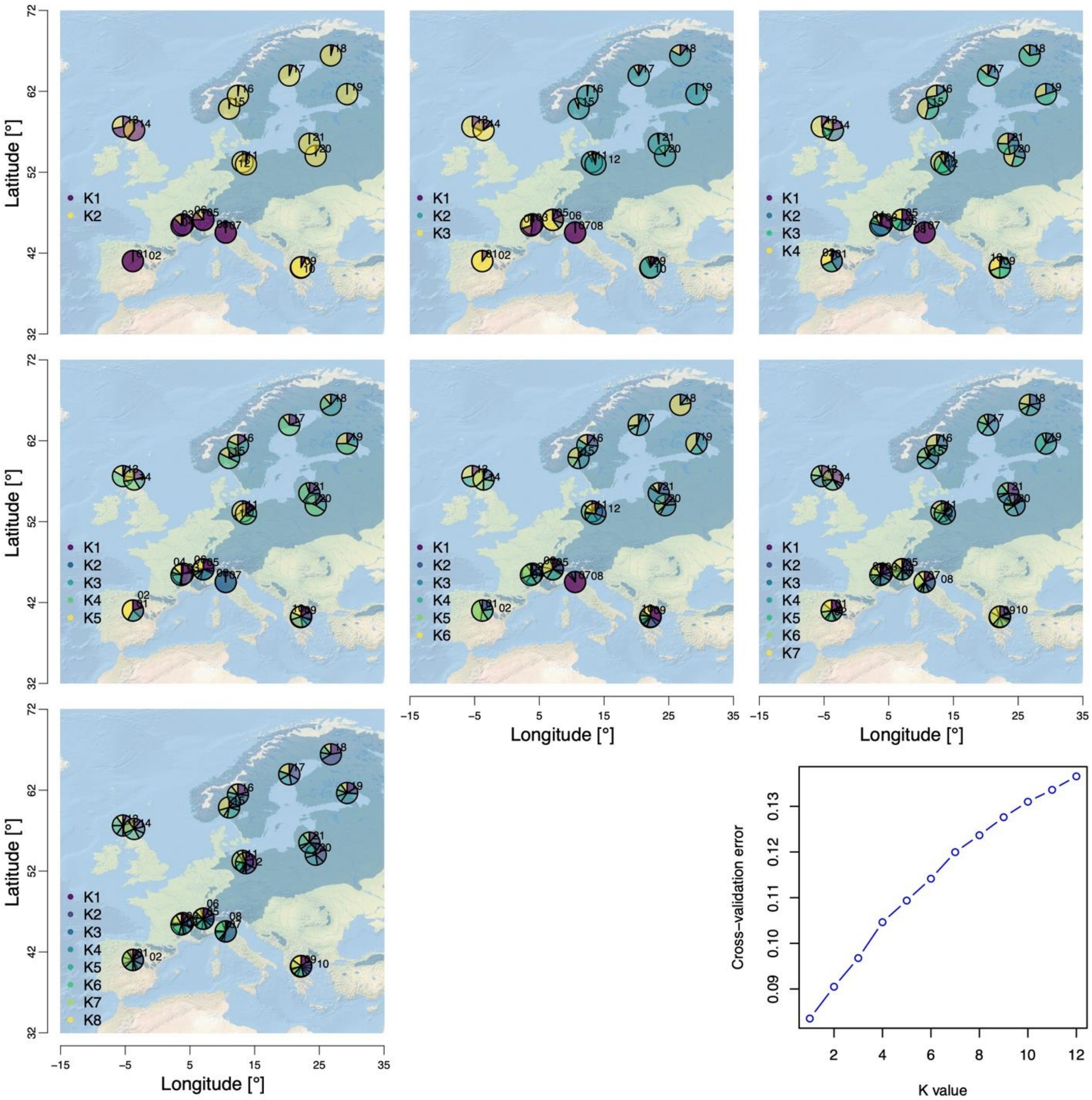

Figure S8. Admixture analysis among populations of *Pinus sylvestris* based on the targeted capture SNP dataset. Geographic distribution[5] of the level of admixture for each population, with colors in pie charts reflecting average assignment probabilities to the respective genetic group (*K* = 2–8). Population codes are explained in Supplementary Data 1. The variation in cross-validation error across *K* values is represented in the bottom right panel, with the lowest value being the optimal number of genetic groups. Source data are provided as a Source Data file-1.

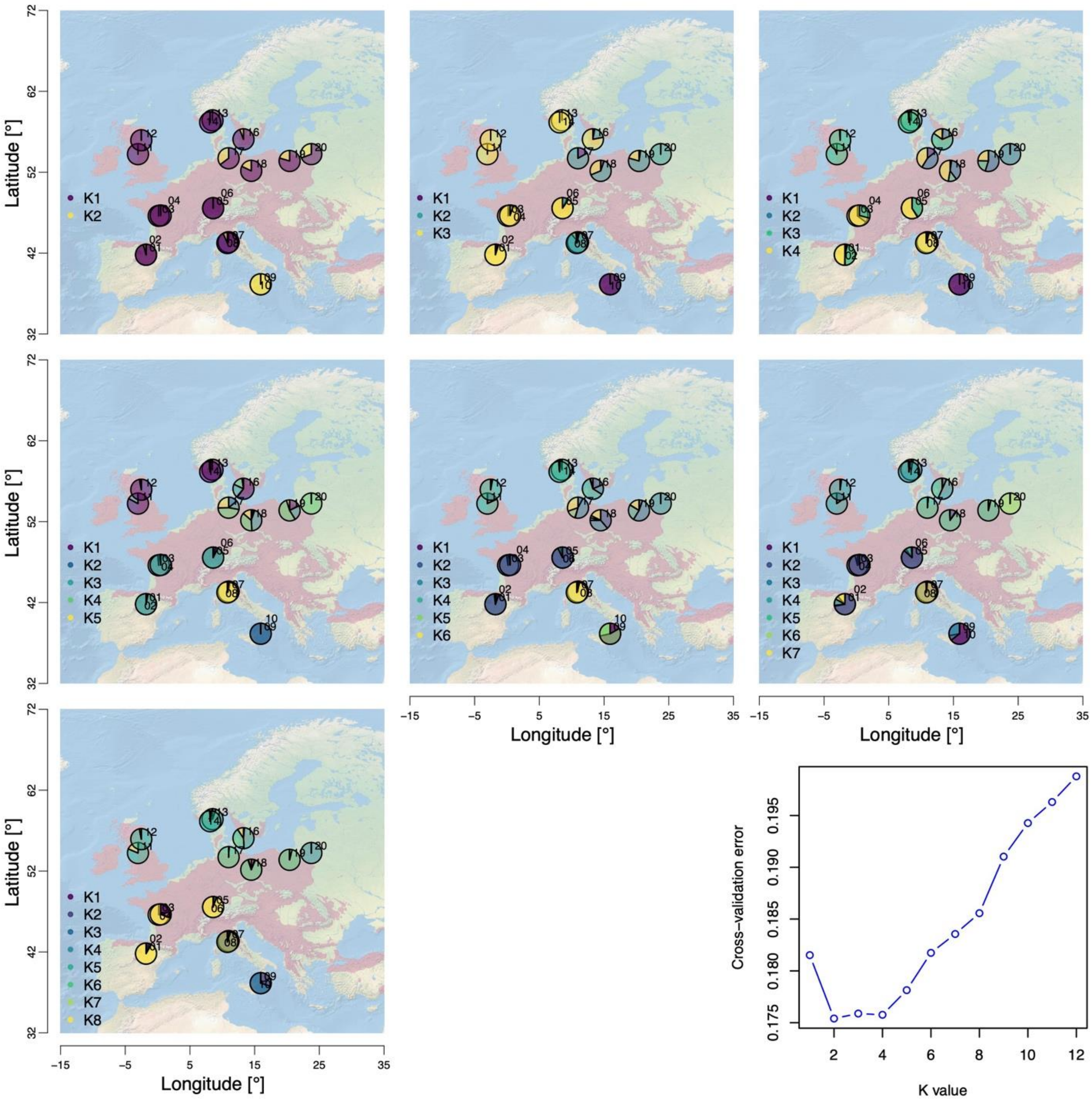

Figure S9. Admixture analysis among populations of *Quercus petraea* based on the targeted capture SNP dataset. Geographic distribution[5] of the level of admixture for each population, with colors in pie charts reflecting average assignment probabilities to the respective genetic group (K = 2–8). Population codes are explained in Supplementary Data 1. The variation in cross-validation error across K values is represented in the bottom right panel, with the lowest value being the optimal number of genetic groups. Source data are provided as a Source Data file-1.

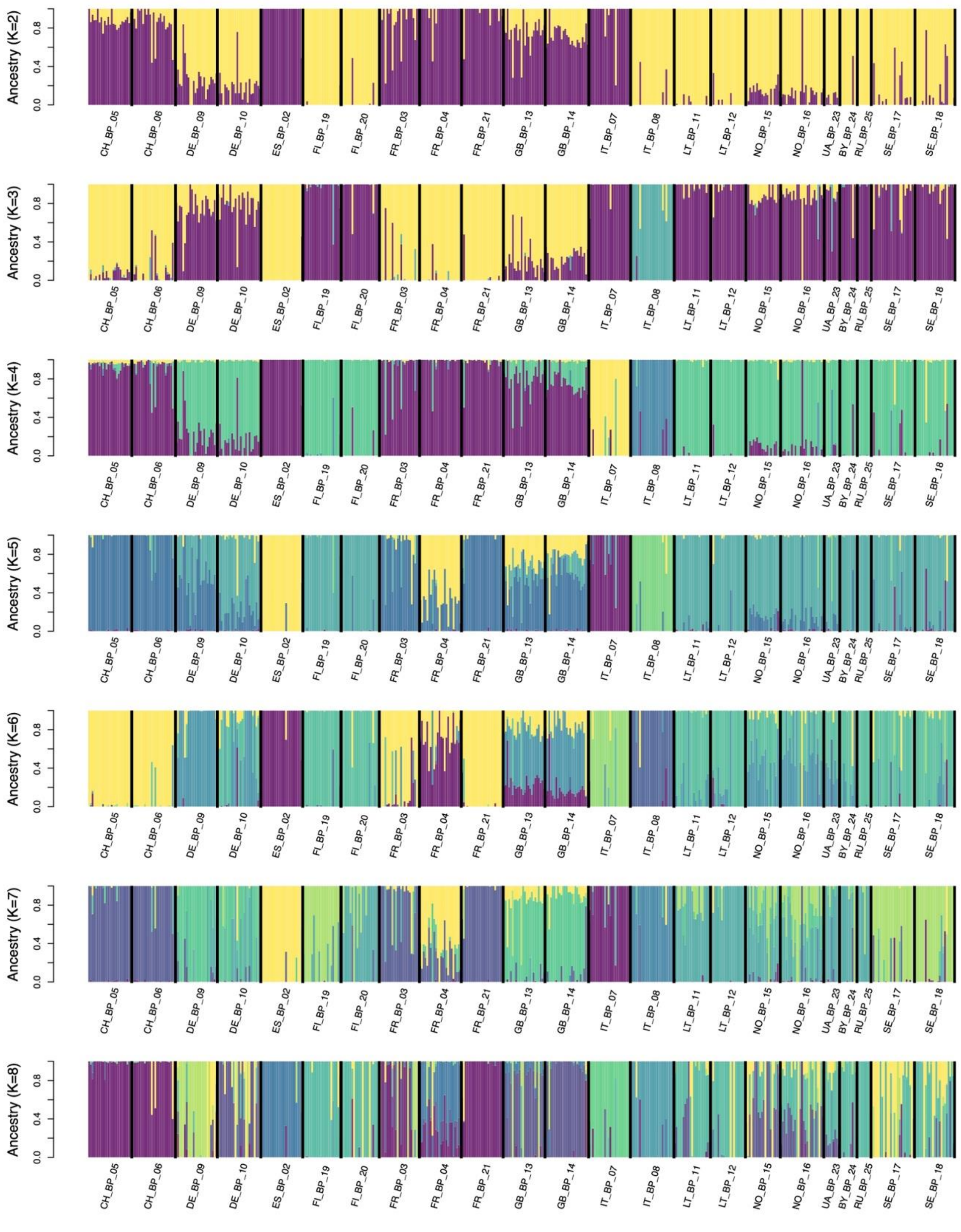

Figure S10. Level of admixture within and among individuals of *Betula pendula* based on the targeted capture SNP dataset. Colors reflect assignment probabilities (i.e. *Q* scores) to respective genetic group (K = 2–8). Population codes are explained in Supplementary Data 1. Singletons were excluded in the admixture analysis. Source data are provided as a Source Data file-1.

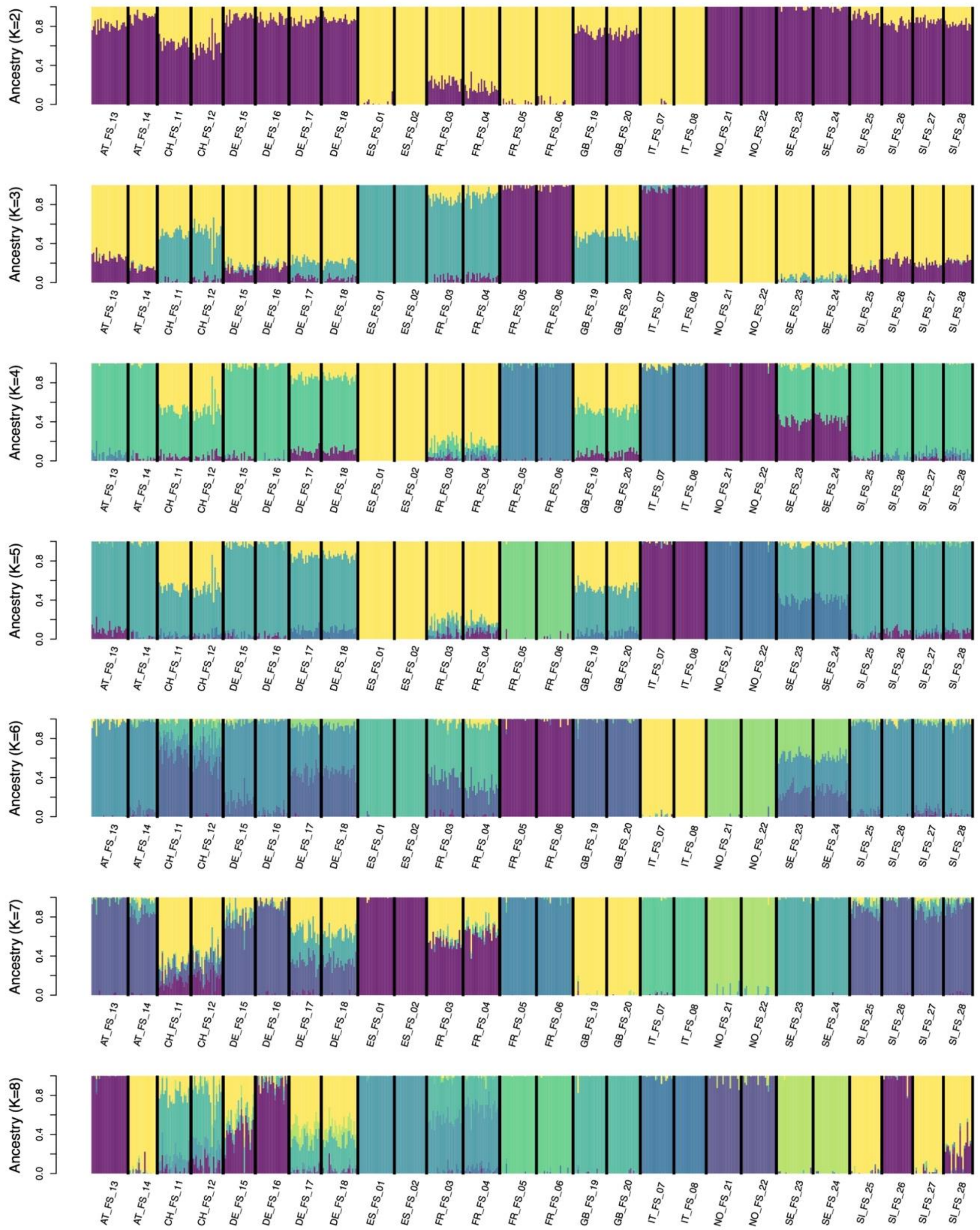

Figure S11. Level of admixture within and among individuals of *Fagus sylvatica* based on the targeted capture SNP dataset. Colors reflect assignment probabilities (i.e. *Q* scores) to respective genetic group ($K = 2–8$). Population codes are explained in Supplementary Data 1. Singletons were excluded in the admixture analysis. Source data are provided as a Source Data file-1.

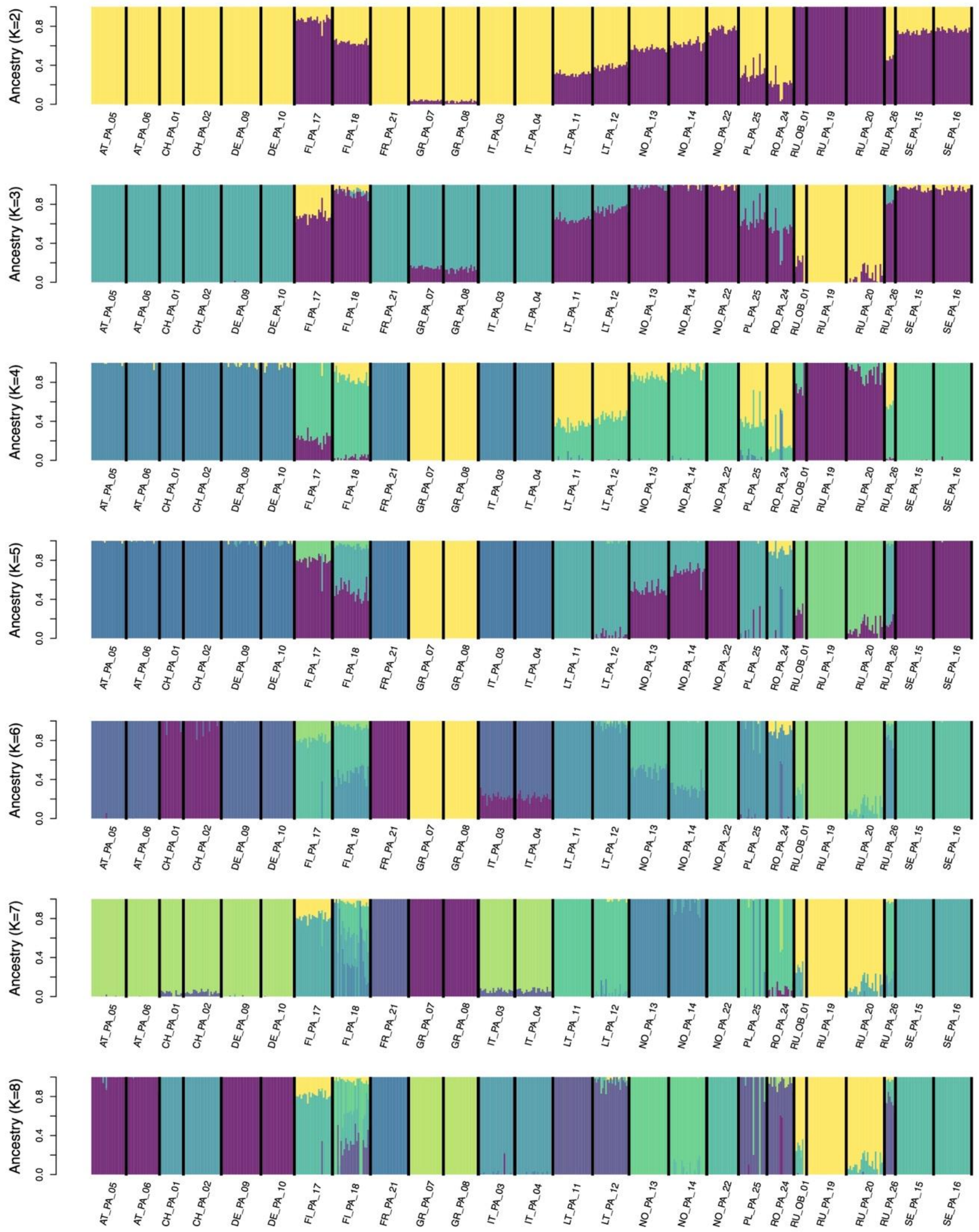

Figure S12. Level of admixture within and among individuals of *Picea abies* based on the targeted capture SNP dataset. Colors reflect assignment probabilities (i.e. *Q* scores) to respective genetic group (*K* = 2–8). Population codes are explained in Supplementary Data 1. Source data are provided as a Source Data file-1.

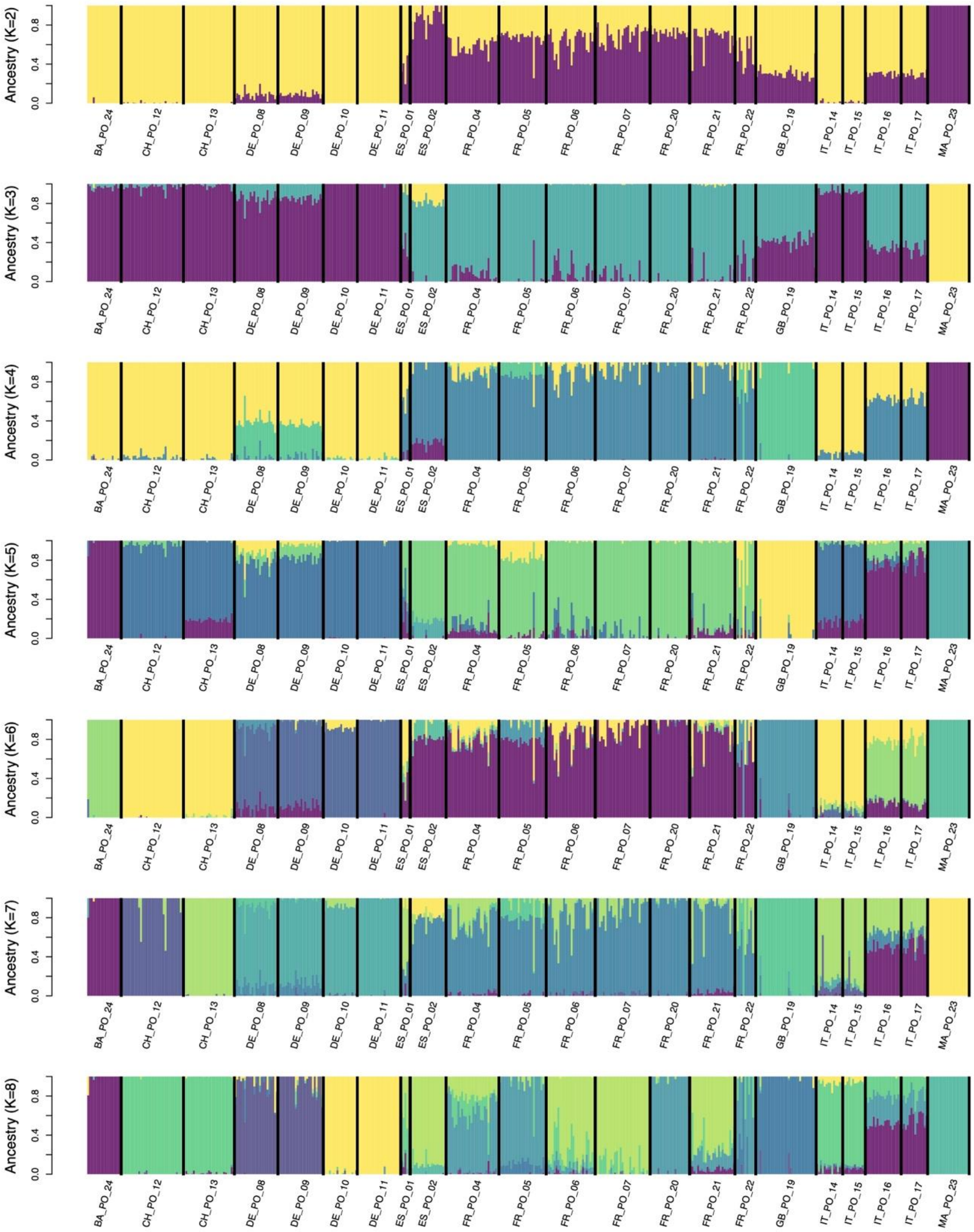

Figure S13. Level of admixture within and among individuals of *Populus nigra* based on the targeted capture SNP dataset. Colors reflect assignment probabilities (i.e. Q scores) to respective genetic group (K = 2–8). Population codes are explained in Supplementary Data 1. Source data are provided as a Source Data file-1.

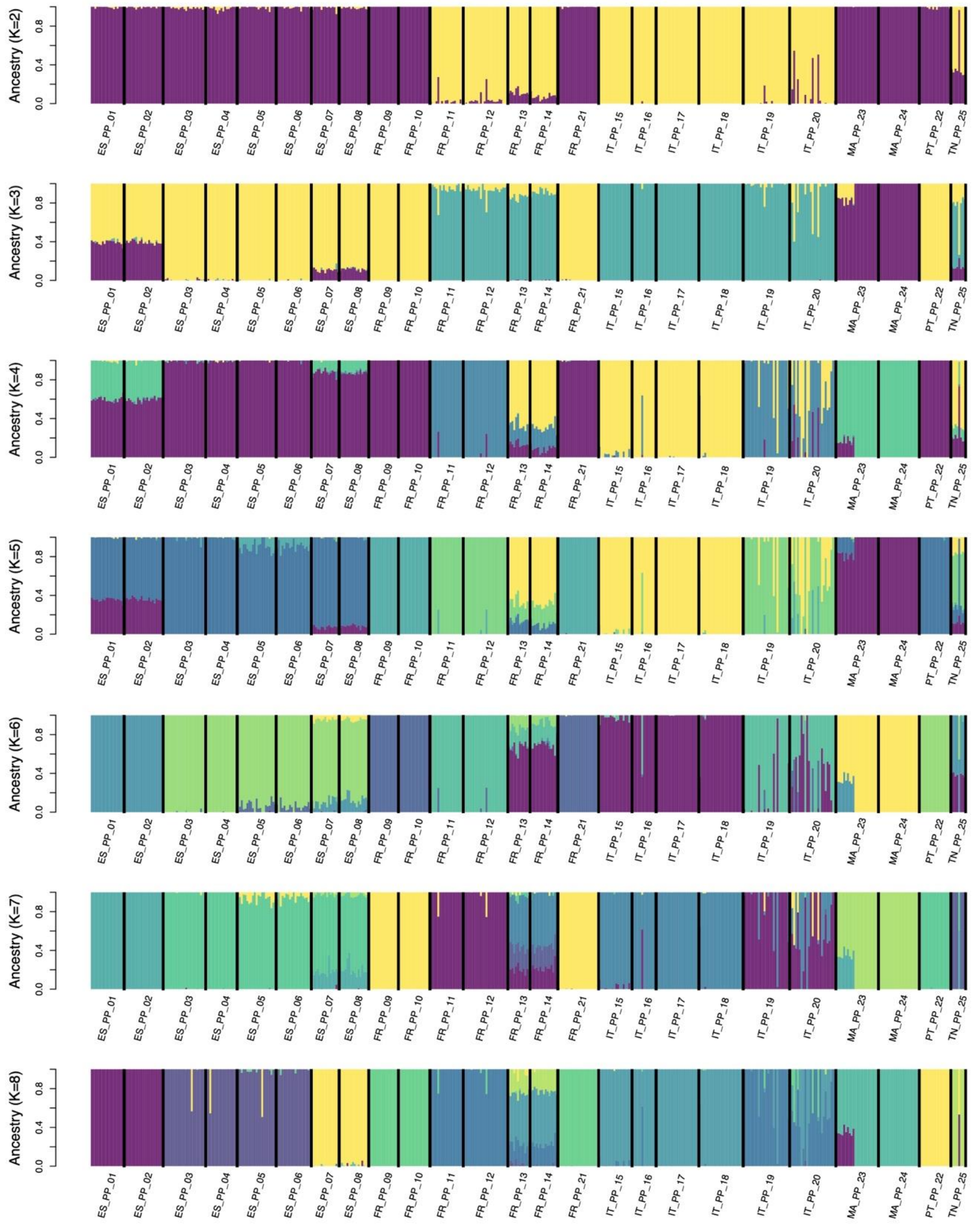

Figure S14. Level of admixture within and among individuals of *Pinus pinaster* based on the targeted capture SNP dataset. Colors reflect assignment probabilities (i.e. Q scores) to respective genetic group (K = 2–8). Population codes are explained in Supplementary Data 1. Source data are provided as a Source Data file-1.

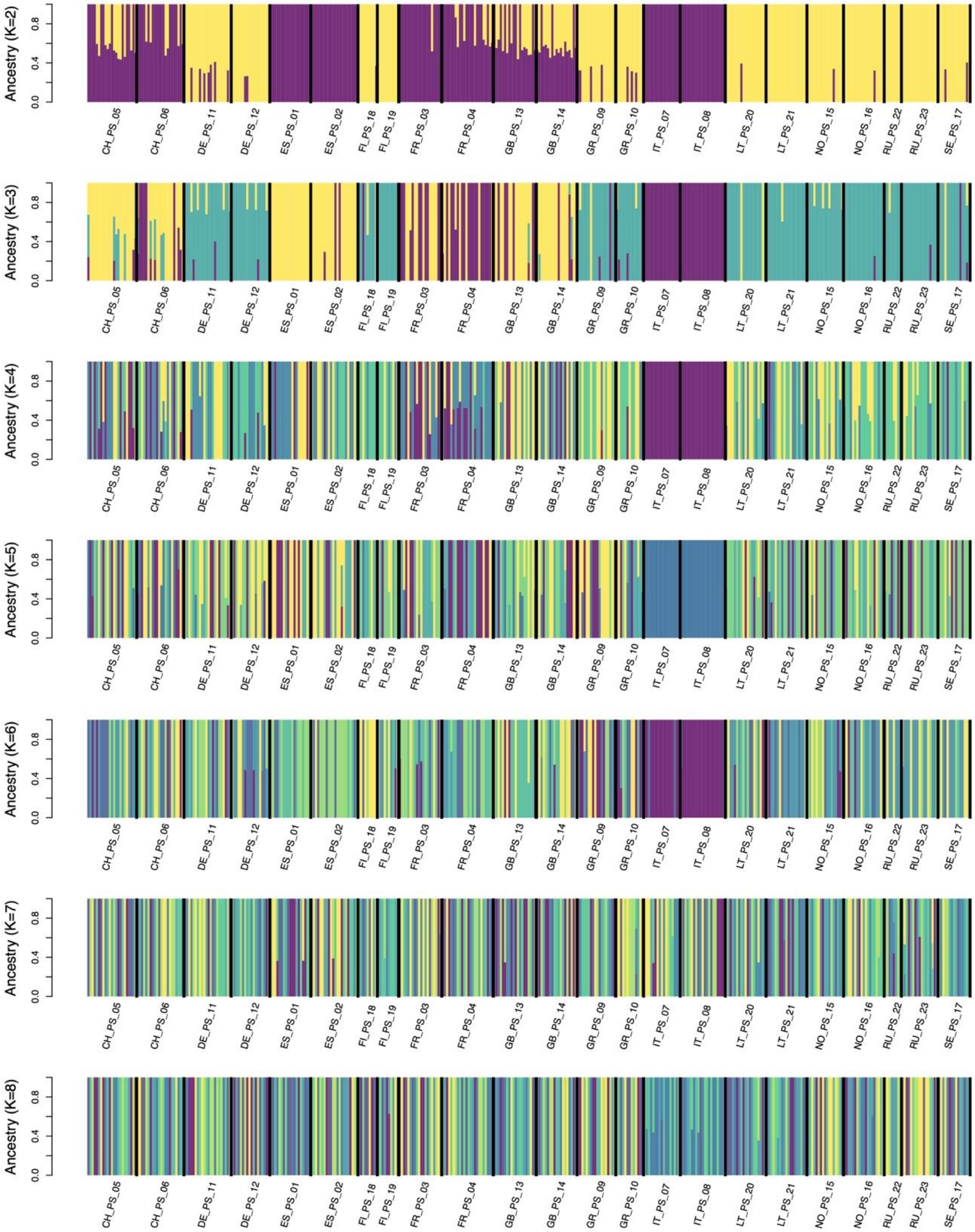

Figure S15. Level of admixture within and among individuals of *Pinus sylvestris* based on the targeted capture SNP dataset. Colors reflect assignment probabilities (i.e. Q scores) to respective genetic group (K = 2–8). Population codes are explained in Supplementary Data 1. Source data are provided as a Source Data file-1.

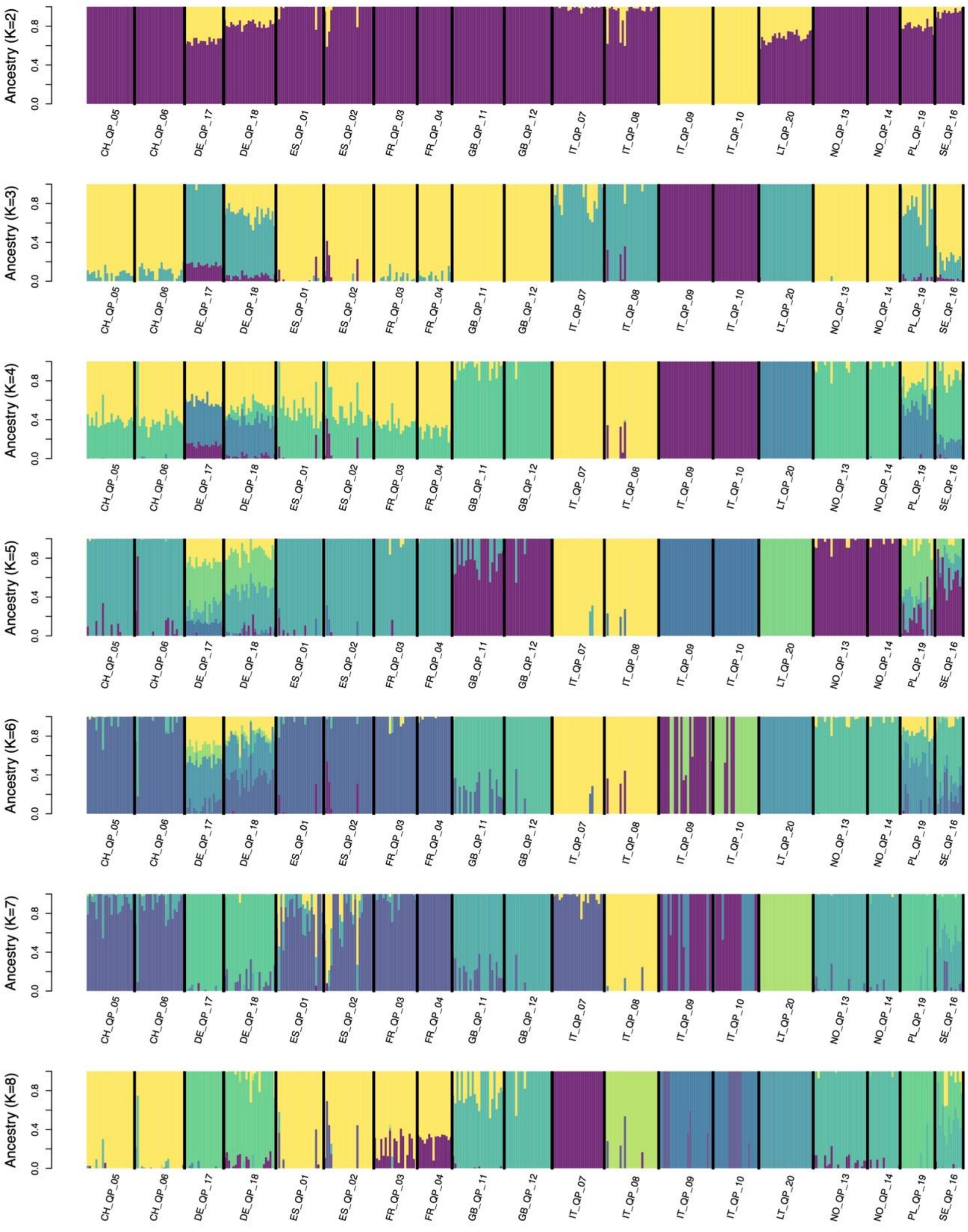

Figure S16. Level of admixture within and among individuals of *Quercus petraea* based on the targeted capture SNP dataset. Colors reflect assignment probabilities (i.e. *Q* scores) to respective genetic group (K = 2–8). Population codes are explained in Supplementary Data 1. Source data are provided as a Source Data file-1.

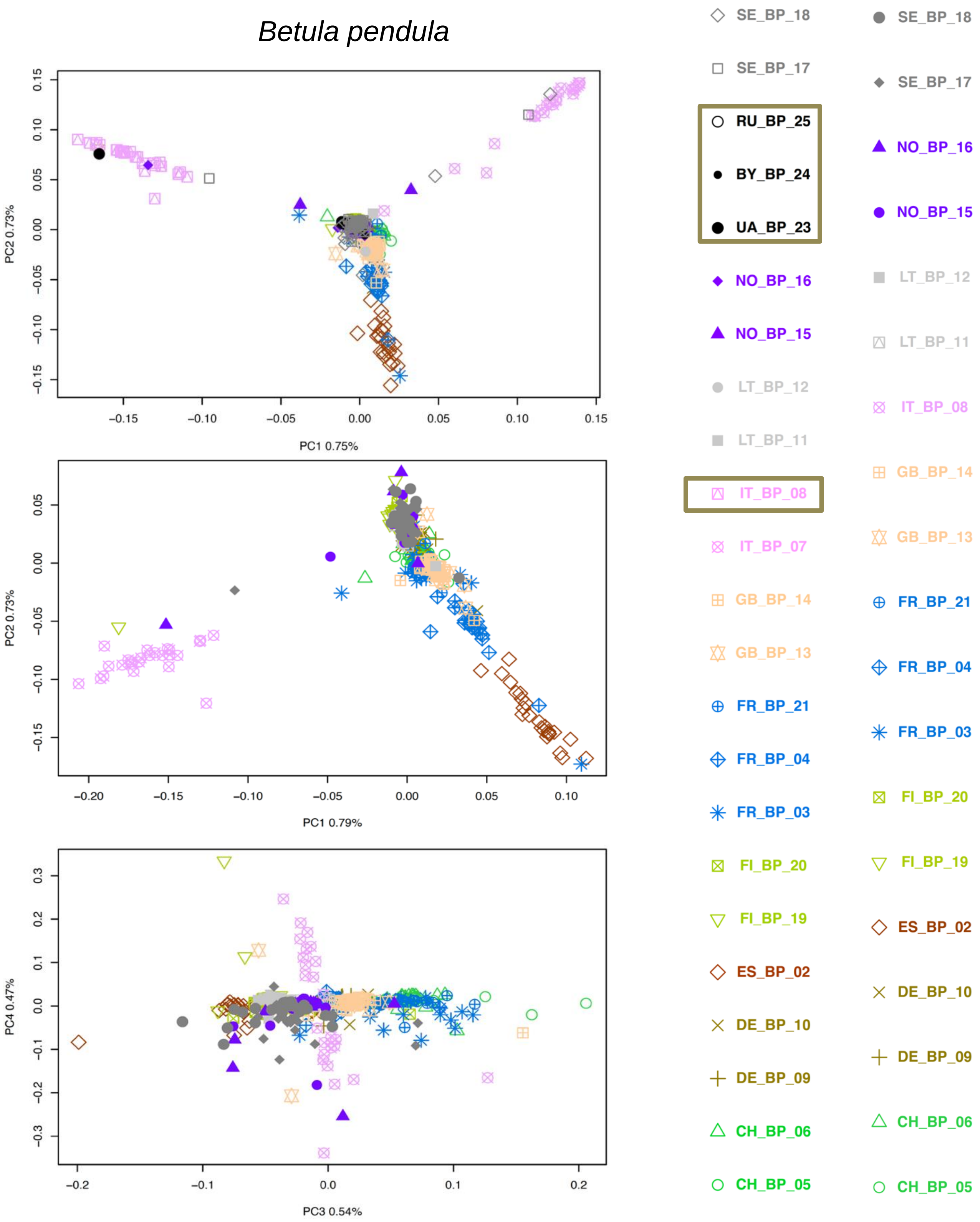

Figure S17. Principal component analysis of population structure of *Betula pendula*. Top panel is for all populations included in SNP set v.5.3. Middle and bottom panels show PC1 and 2 and PC3 and 4 of a PCA excluding outlier populations and individuals from top panel and focusing on Western Europe populations (SNP set v.6.3.1). Completely excluded populations are boxed. Leftmost most legend is for top panel, rightmost is for middle and bottom panels. Source data are provided as a Source Data file-1.

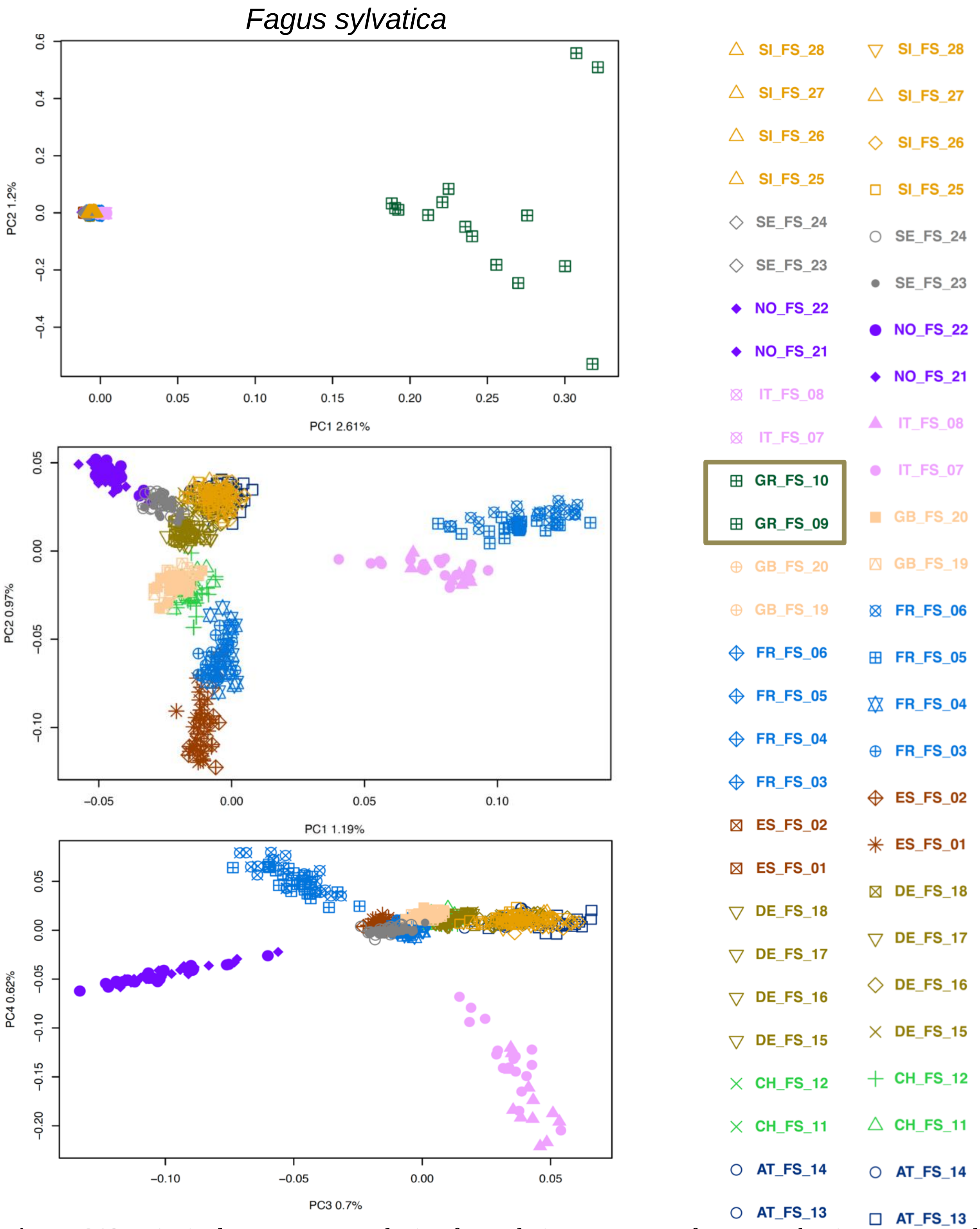

Figure S18. Principal component analysis of population structure of *Fagus sylvatica*. Top panel is for all populations included in SNP set v.5.3. Middle and bottom panels show PC1 and 2 and PC3 and 4 of a PCA excluding outlier populations and individuals from top panel and focusing on Western Europe populations (SNP set v.6.3.1). Completely excluded populations are boxed. Leftmost most legend is for top panel, rightmost is for middle and bottom panels. Source data are provided as a Source Data file-1.

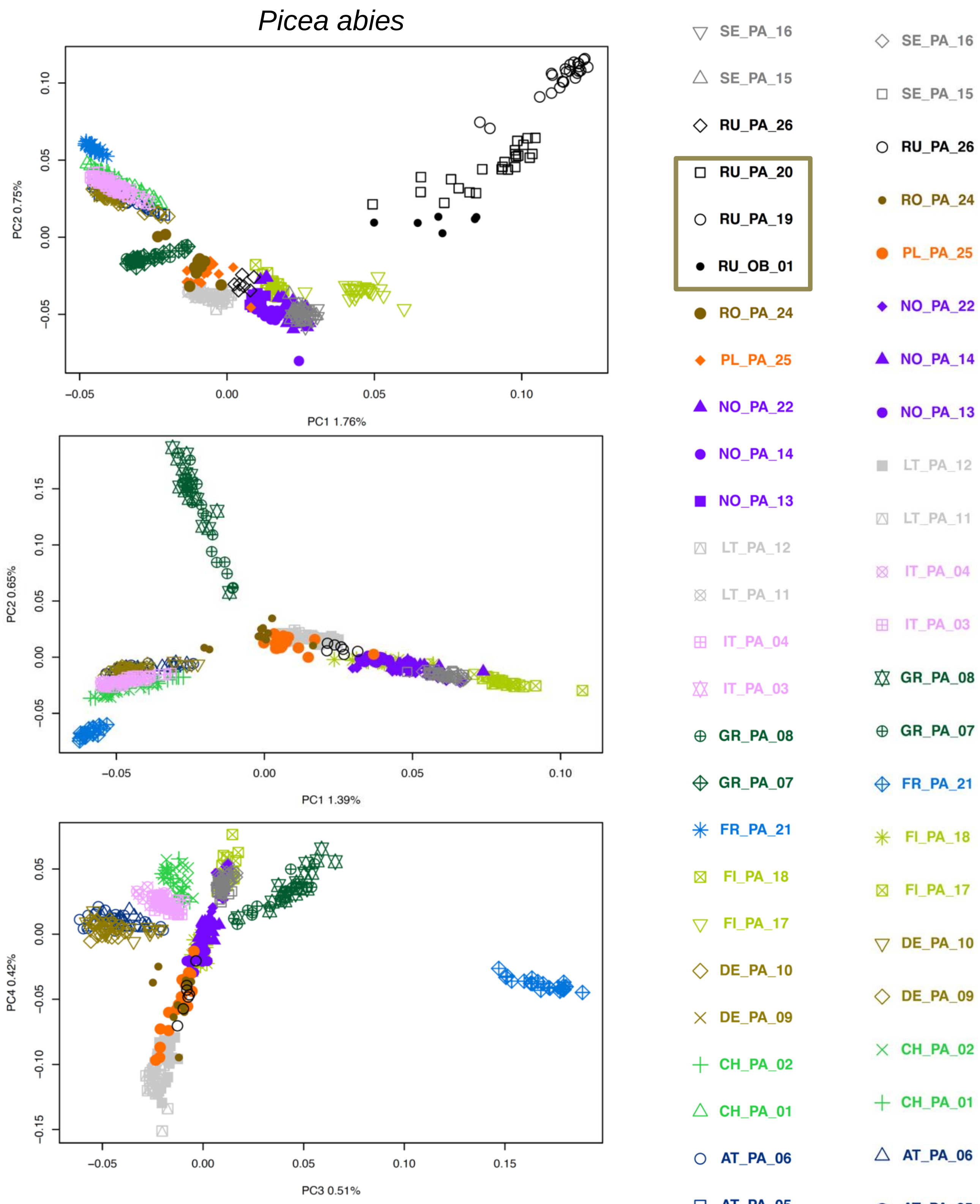

Figure S19. Principal component analysis of population structure of *P*[illegible]. Top pa[illegible] all populations included in SNP set v.5.3. Middle and bottom panels show PC1 and 2 and PC3 and 4 of a PCA excluding outlier populations and individuals from top panel and focusing on Western Europe populations (SNP set v.6.3.1). Completely excluded populations are boxed. Leftmost most legend is for top panel, rightmost is for middle and bottom panels. Source data are provided as a Source Data file-1.

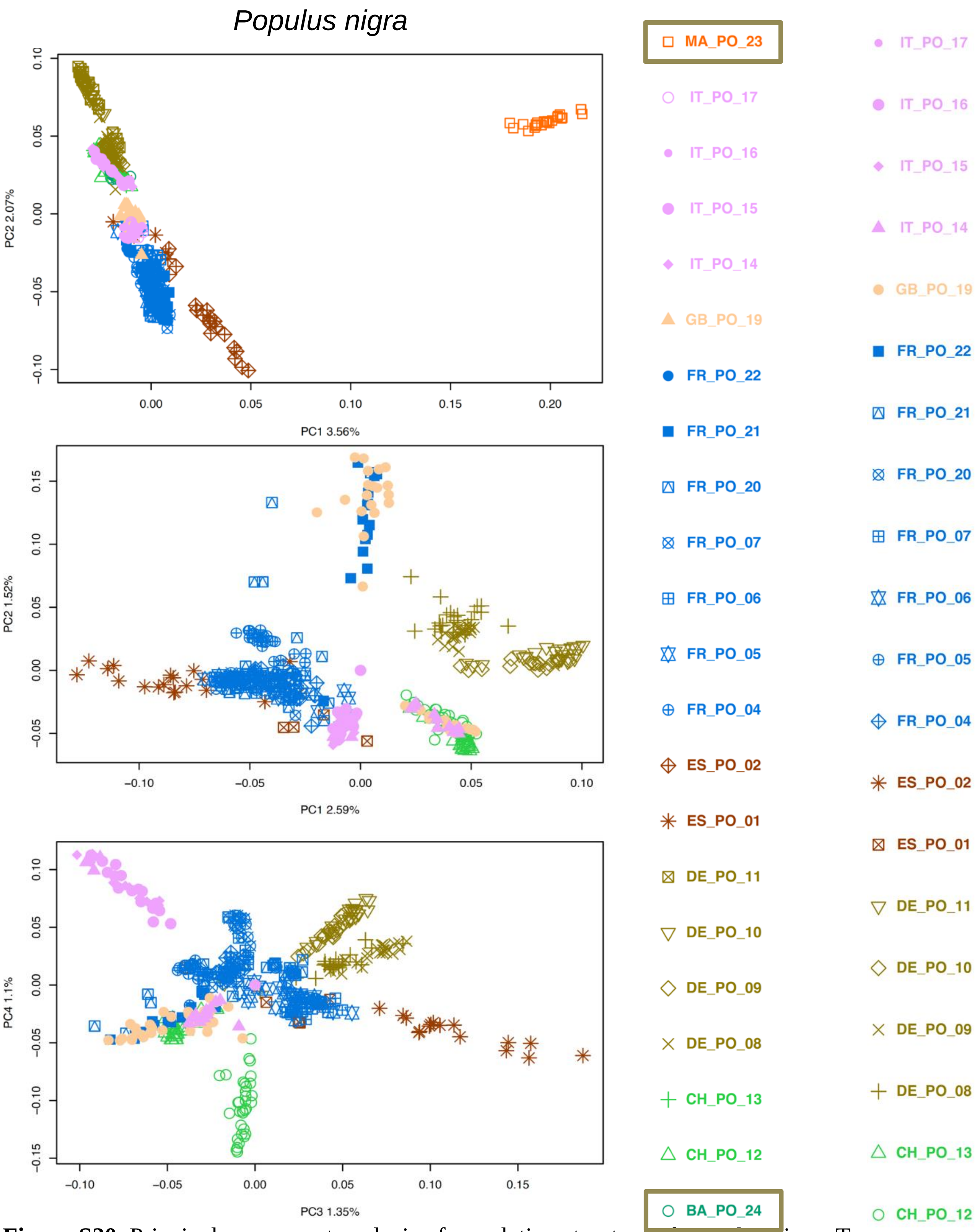

Figure S20. Principal component analysis of population structure of *Populus nigra*. Top panel is for all populations included in SNP set v.5.3. Middle and bottom panels show PC1 and 2 and PC3 and 4 of a PCA excluding outlier populations and individuals from top panel and focusing on Western Europe populations (SNP set v.6.3.1). Completely excluded populations are boxed. Leftmost most legend is for top panel, rightmost is for middle and bottom panels. Source data are provided as a Source Data file-1.

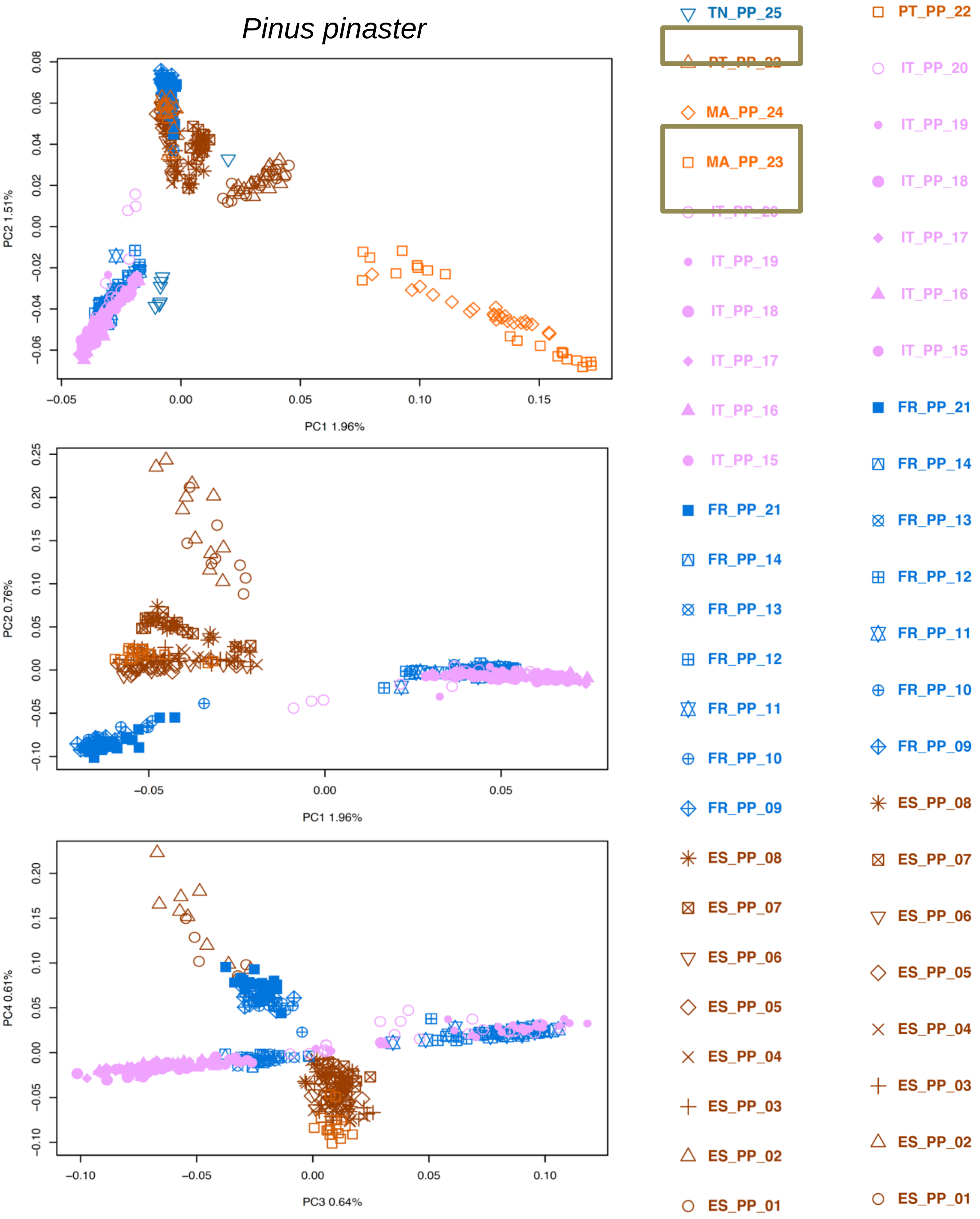

Figure S21. Principal component analysis of population structure of *Pinus pinaster*. Top panel is for all populations included in SNP set v.5.3. Middle and bottom panels show PC1 and 2 and PC3 and 4 of a PCA excluding outlier populations and individuals from top panel and focusing on Western Europe populations (SNP set v.6.3.1). Completely excluded populations are boxed. Leftmost most legend is for top panel, rightmost is for middle and bottom panels. Source data are provided as a Source Data file-1.

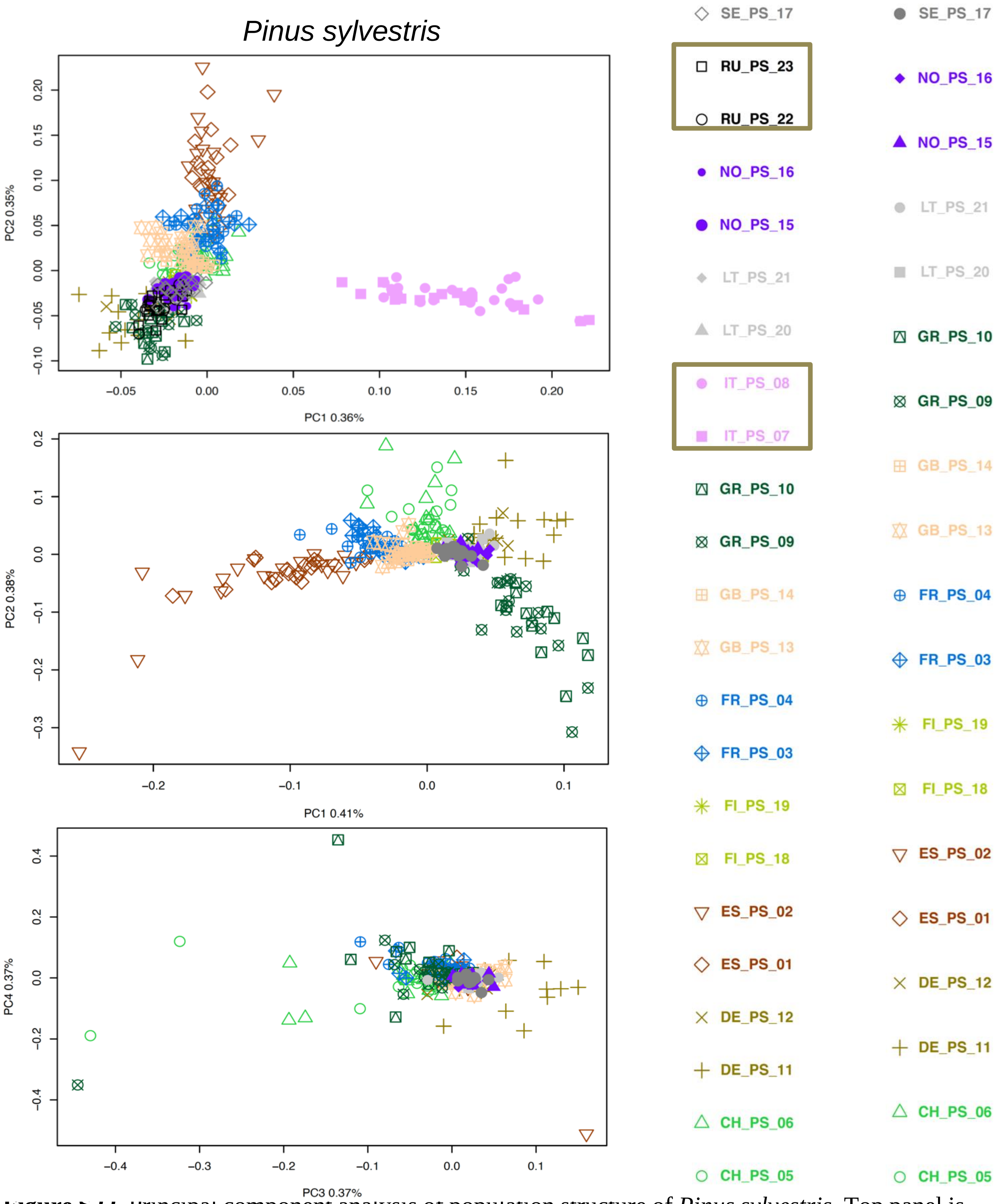

Figure S22. Principal component analysis of population structure of *Pinus sylvestris*. Top panel is for all populations included in SNP set v.5.3. Middle and bottom panels show PC1 and 2 and PC3 and 4 of a PCA excluding outlier populations and individuals from top panel and focusing on Western Europe populations (SNP set v.6.3.1). Completely excluded populations are boxed. Leftmost most legend is for top panel, rightmost is for middle and bottom panels. Source data are provided as a Source Data file-1.

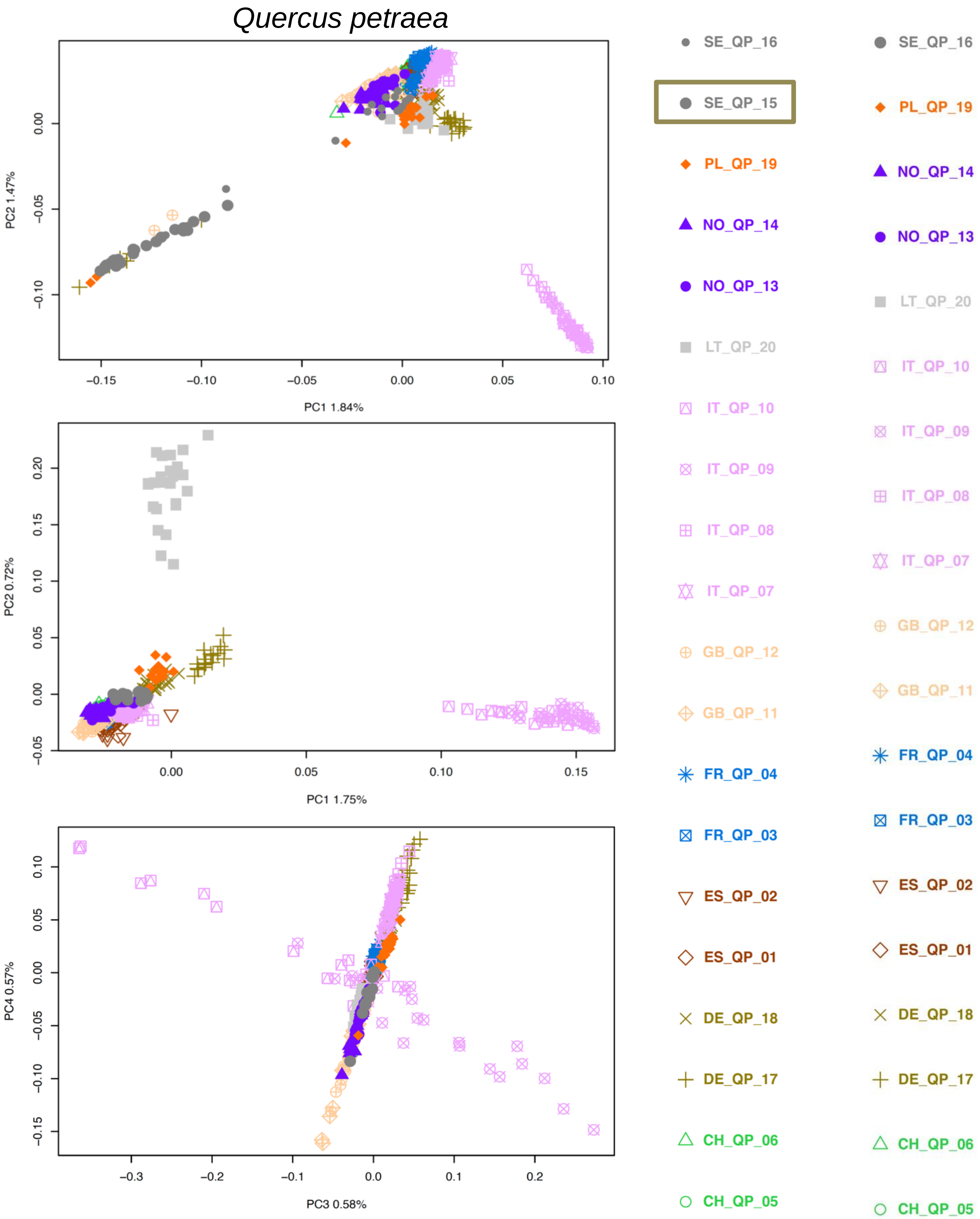

Figure S23. Principal component analysis of population structure of *Quercus petraea*. Top panel is for all populations included in SNP set v.5.3. Middle and bottom panels show PC1 and 2 and PC3 and 4 of a PCA excluding outlier populations and individuals from top panel and focusing on Western Europe populations (SNP set v.6.3.1). Completely excluded populations are boxed. Leftmost most legend is for top panel, rightmost is for middle and bottom panels. Source data are provided as a Source Data file-1.

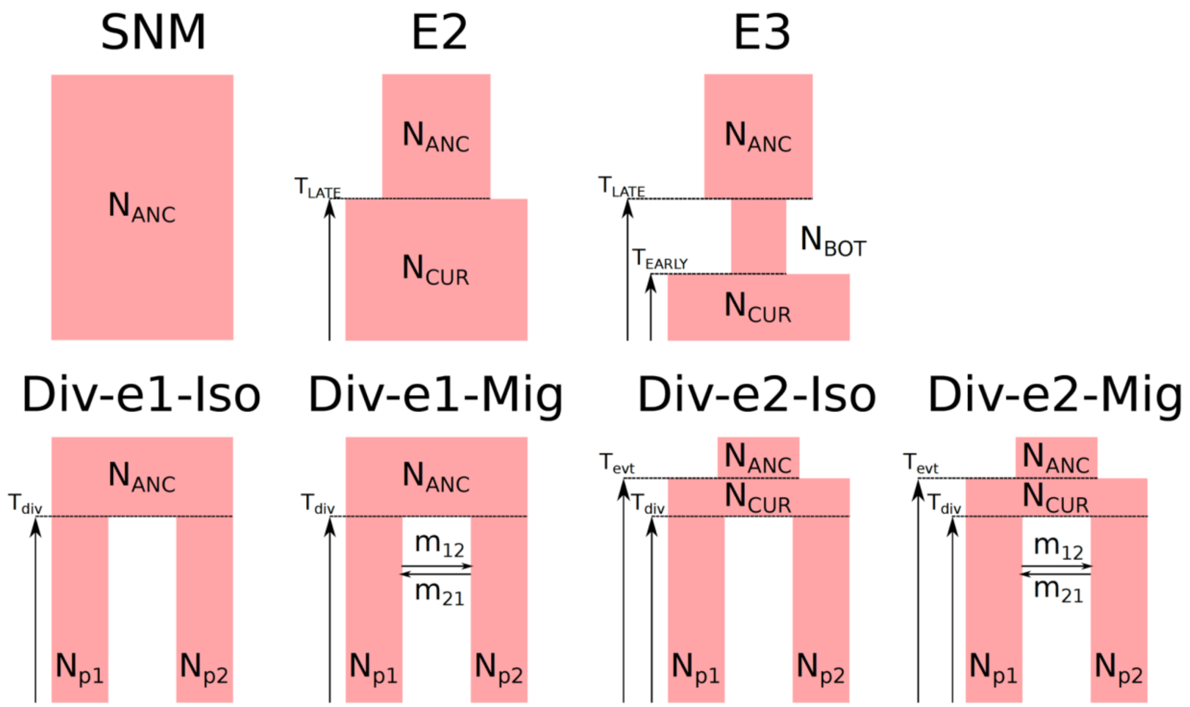

Figure S24. Schematics of the Fastsimcoal 2 models used in this study and their parameters. The blocks represent the evolution of populations with their lengths along the horizontal axis reflecting the population size and the time on the vertical axis from past (top) to present (bottom). Model abbreviations: SNM = Standard Neutral Model, E2 = 2 epoch model, E3 = 3 epoch model, Div-e1-Iso and Div-e2-Iso = Divergence model of two isolated populations both deriving from the same ancestral population that didn't or did experience a single demographic event in the past respectively, Div-e1-Mig and Div-e2-Mig = Divergence model of two populations with migration between both deriving from the same ancestral population that didn't or did experience a single demographic event in the past respectively. Parameter abbreviations: N_{ANC}, N_{CUR}, N_{BOT}, N_{p1}, N_{p2}: effective population size of respectively the ancestral, current, bottleneck, diverged population 1 or diverged population 2. T_{LATE} T_{EARLY}: time of the latest or earliest (backward in time) demographic event. T_{evt} and T_{div}: time of the demographic or divergence event. m_{12} m_{21}: migration rate from population 1 to 2 and 2 to 1 respectively (backward in time).

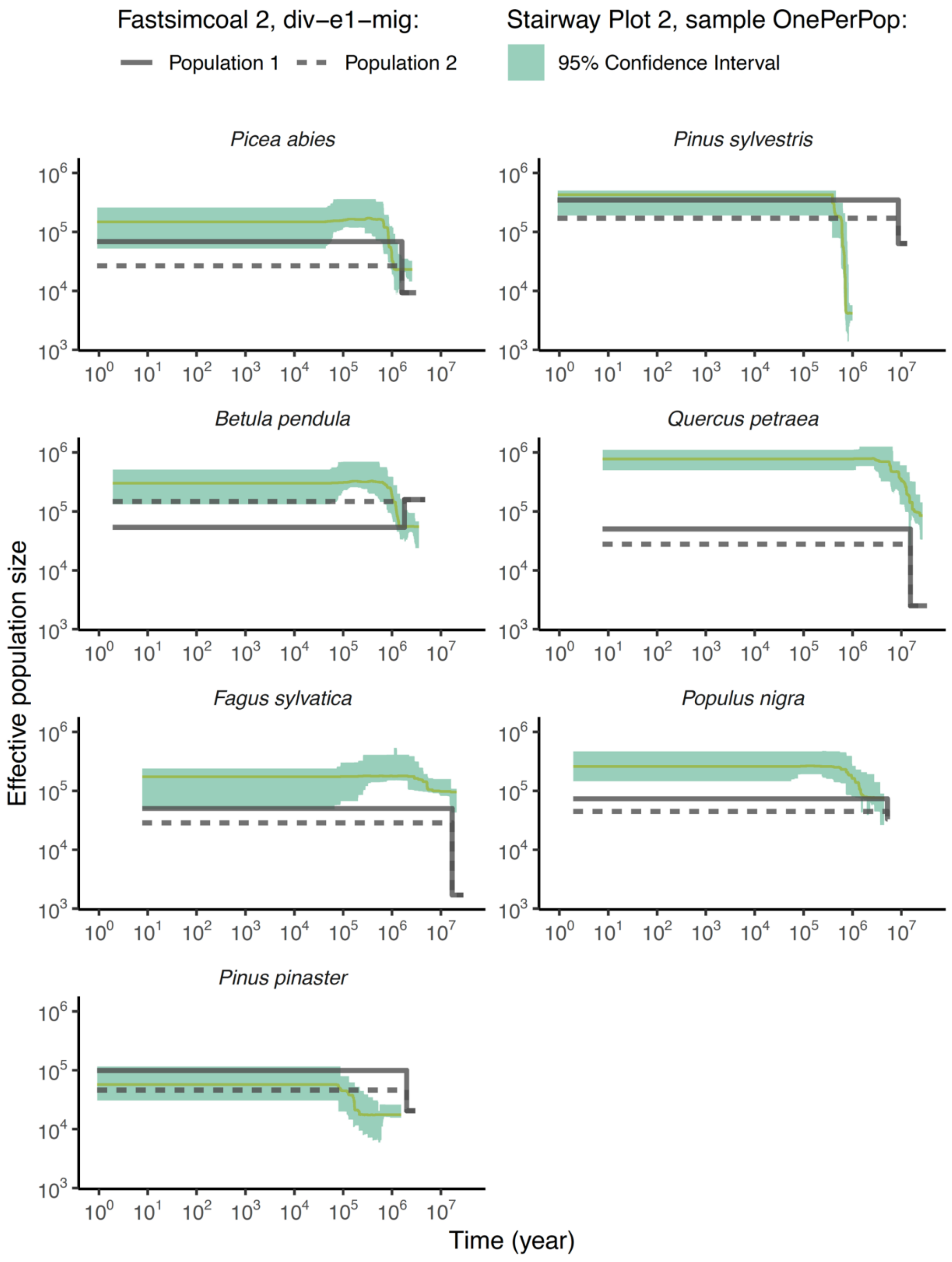

Figure S25. Comparisons of the results obtained with Stairway Plot 2 and the divergence models (div-e1-mig) implemented with fastsimcoal2. Source data are provided as a Source Data file-2.

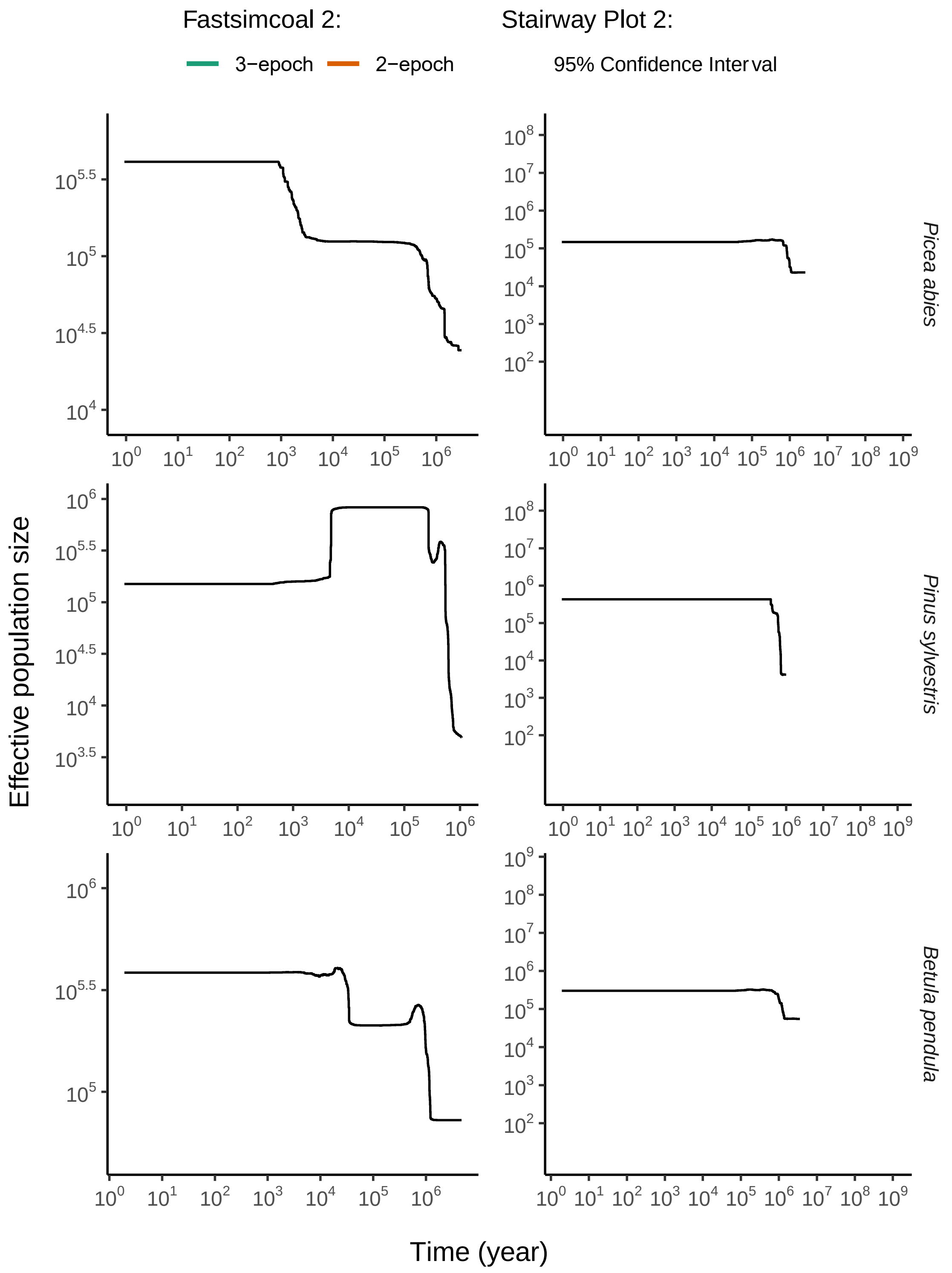

Fastsimcoal 2:
3−epoch
2−epoch
Stairway Plot 2:
95% Confidence Interval
Effective population size
Time (year)
Picea abies
Pinus sylvestris
Betula pendula

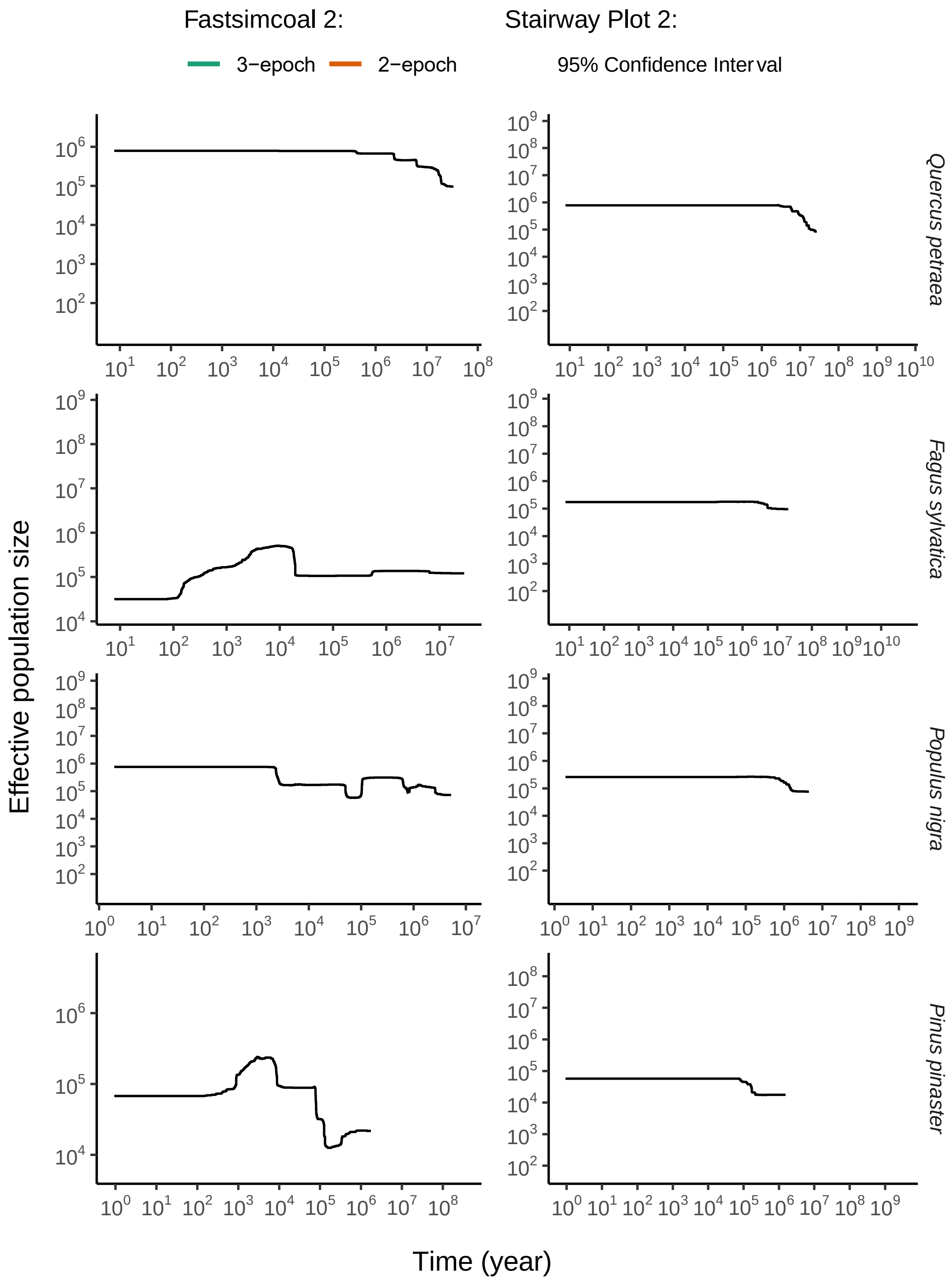

Figure S26. Comparison of the results obtained for the demographic dynamics of the seven forest tree species using two different sampling sets and three approaches. More specifically, results are presented for the Stairway Plot 2 model (in yellow) and for two demographic models tested using fastsimcoal2, i.e. 2-epoch (in red) and 3-epoch (in green), which allow one and two demographic events in the population, respectively. The analyses were performed using the site frequency spectrum computed either over all samples left panel) or over a random subset of one haplotype per location (OneperPop, right panel). Source data are provided as a Source Data file-2.

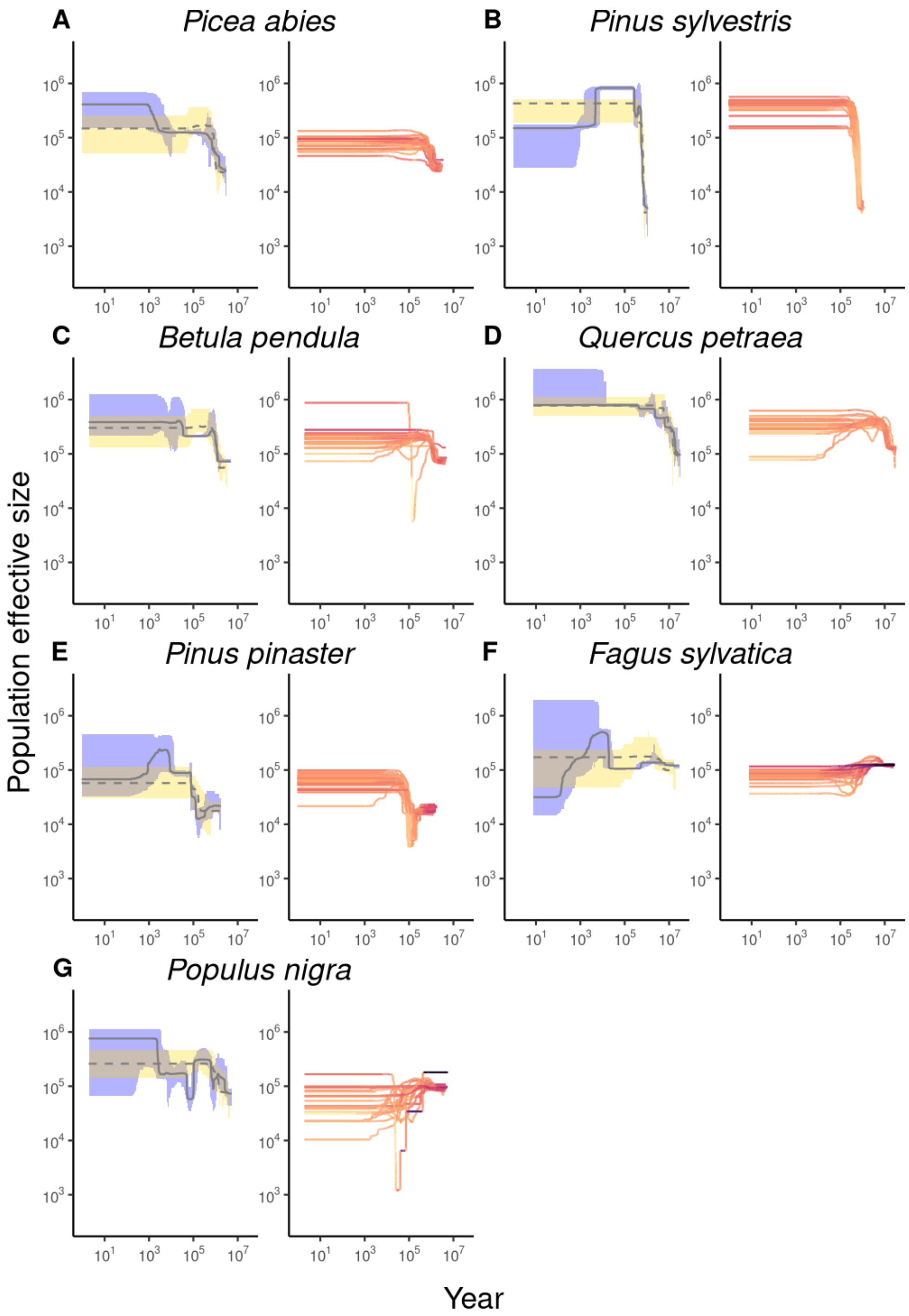

Figure S27. Stairway Plot 2 results for each species and across different sampling designs. For each species, results at the global and population levels are presented. At the global level (left plot in each panel), all-sample results (in blue) and one-per-population results (in yellow) are represented, with a line for the median level and a band for the 95% confidence interval. At the population level (right plot in each panel), each colored line represents the median result of the Stairway Plot 2 analysis run on a single population. Lighter to darker line colors represent areas with larger to smaller confidence intervals. Source data are provided as a Source Data file-3.

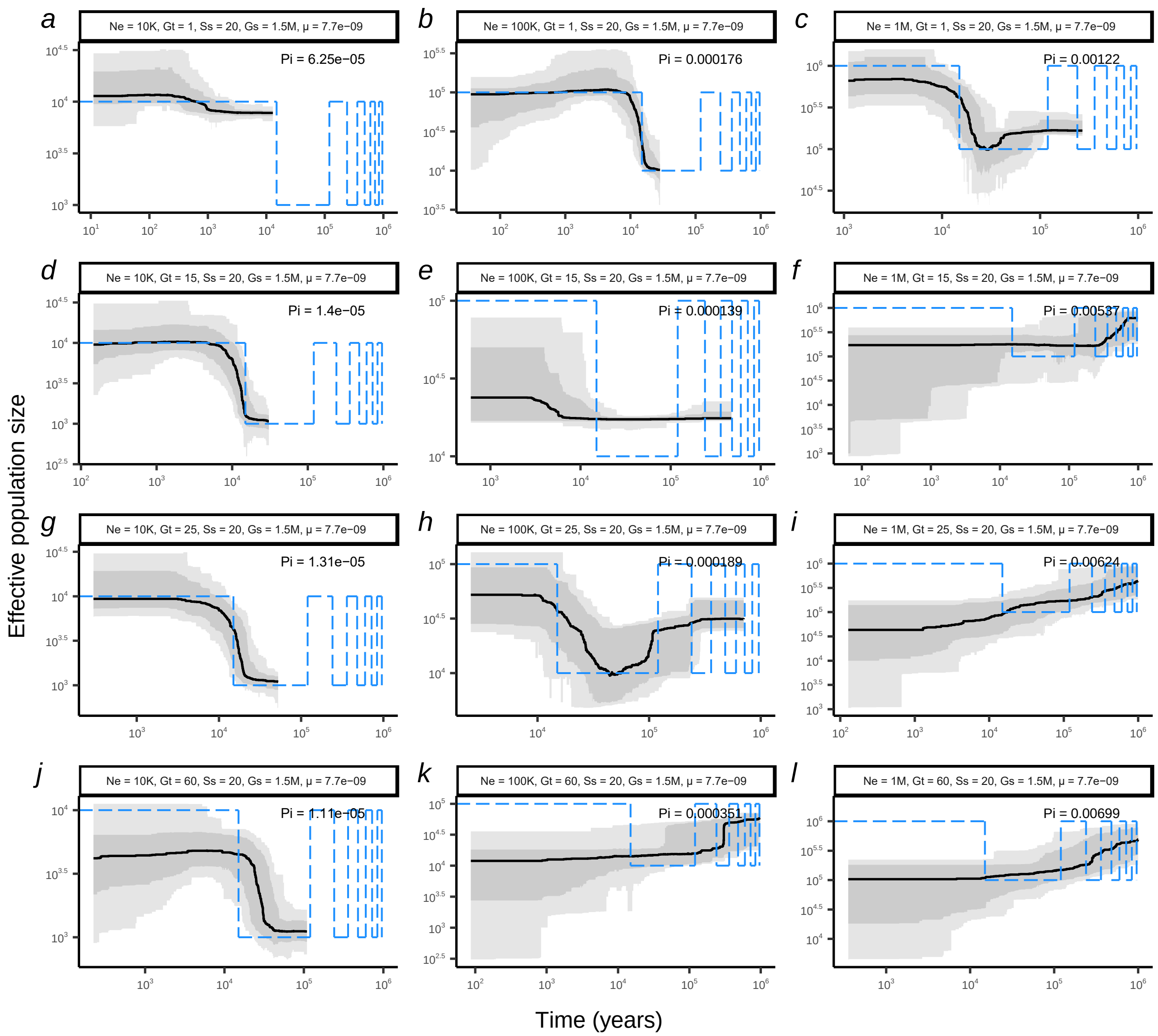

Figure S28. Stairway plot 2 inference of the change of effective population size (N_e) over time (in years, from present to past) of an oscillating population. The black lines represent the median estimates (over 200 simulations), dark and light shades are respectively the 95% and 99% confidence intervals. The blue and dashed line represent the theoretical model simulated with *Fastsimcoal2*. Each panel corresponds to a different starting N_e and generation time (Gt). Across simulations, 20 haploid genomes were simulated with a sample size of the genome of 1.5 Mbp (15K contigs of length 100 bp) and a mutation rate of 7.7×10^{-9} per site per generation. Source data are provided as a Source Data file-4.

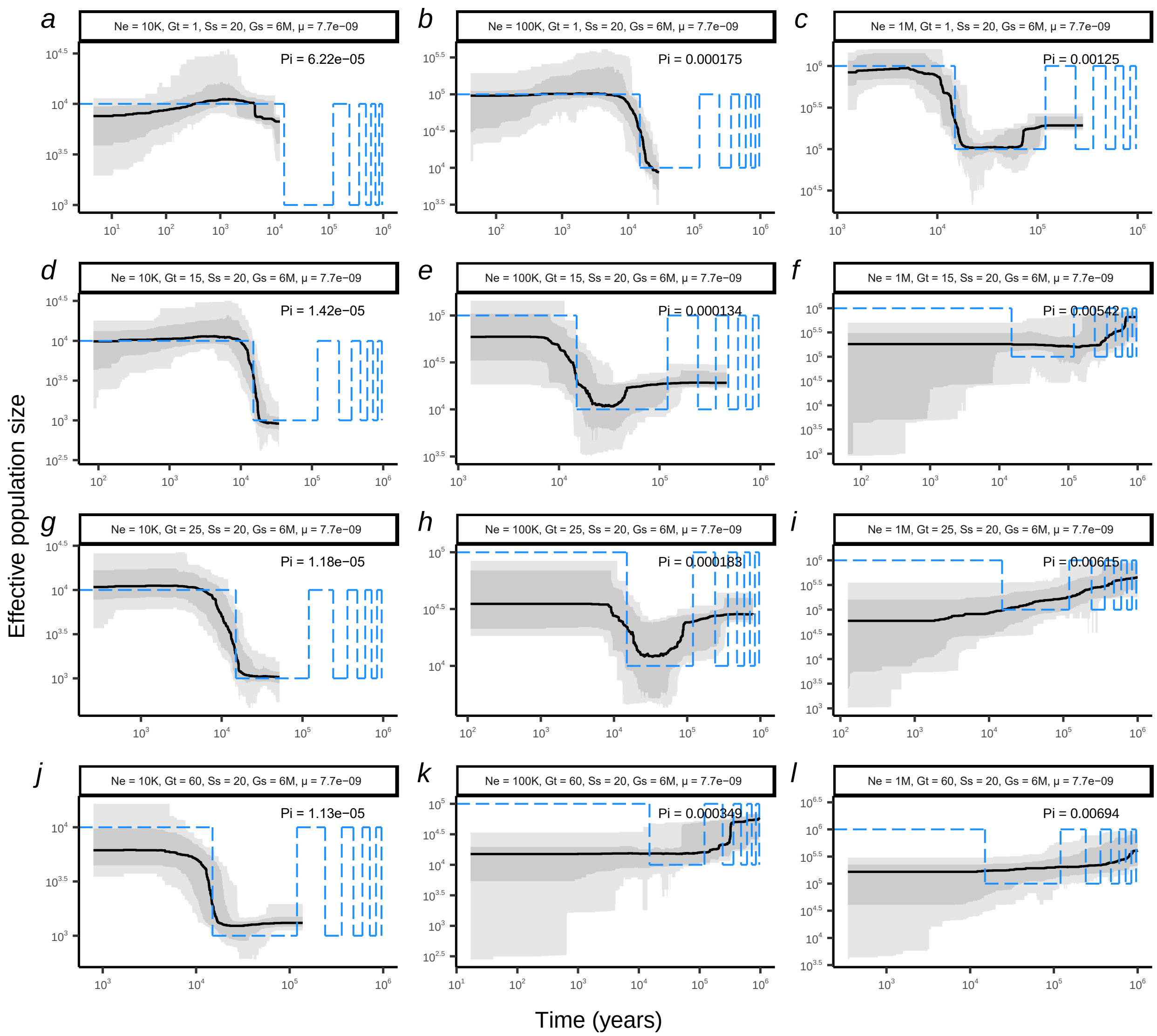

Figure S29. Same as in Fig. S28, but simulations with sample size (Ss) of 20 haploid genomes, with genome size (Gs) of 6 Mbp (60K contigs of length 100 bp) and a mutation rate of 7.7×10^{-9} per site per generation. Source data are provided as a Source Data file-4.

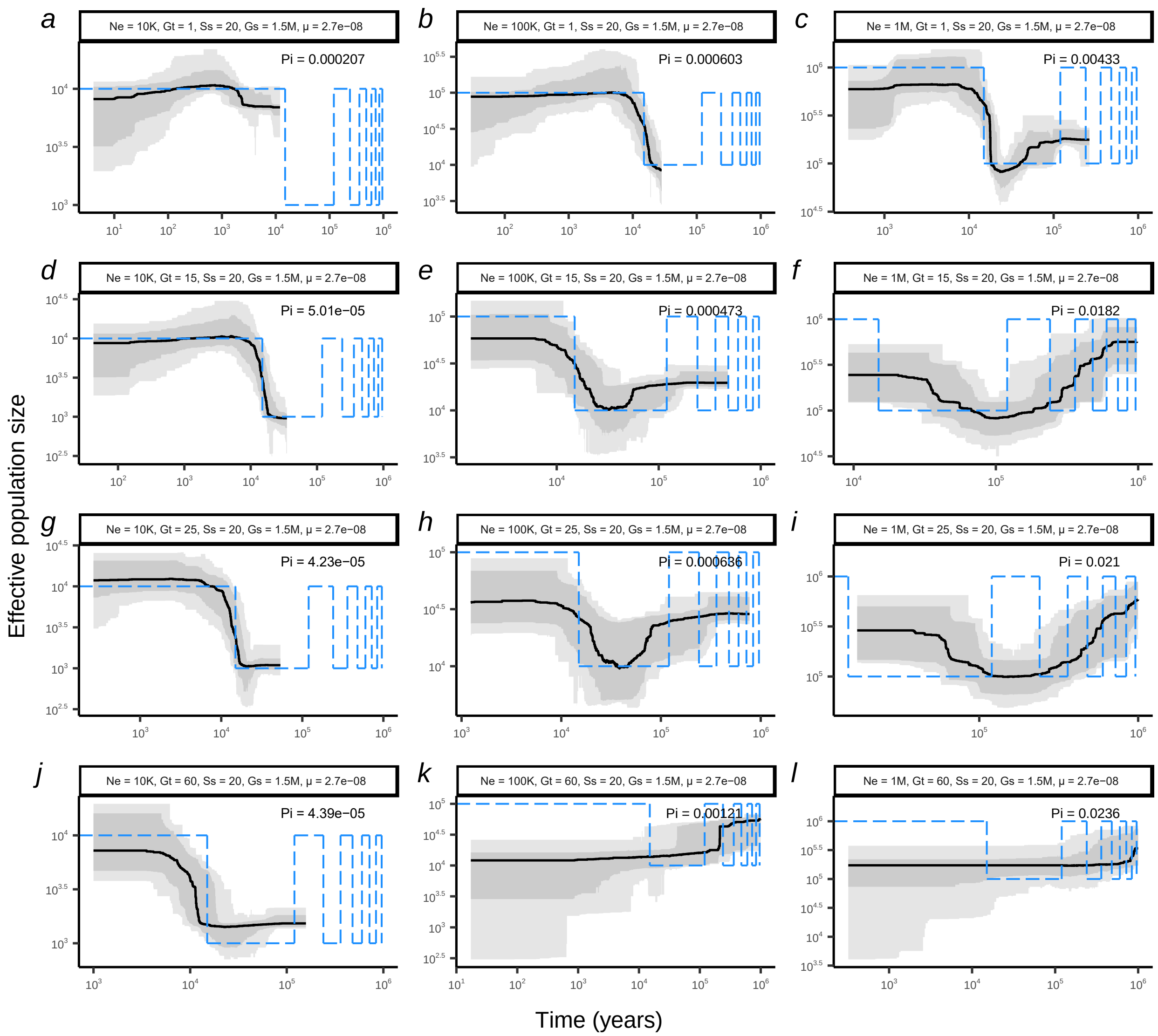

Figure S30. Same as in Fig. S28 but simulations with sample size (Ss) of 20 haploid genomes, with genome size (Gs) of 1.5 Mbp (15K contigs of length 100 bp) and a mutation rate of 2.7×10^{-8} per site per generation. Source data are provided as a Source Data file-4.

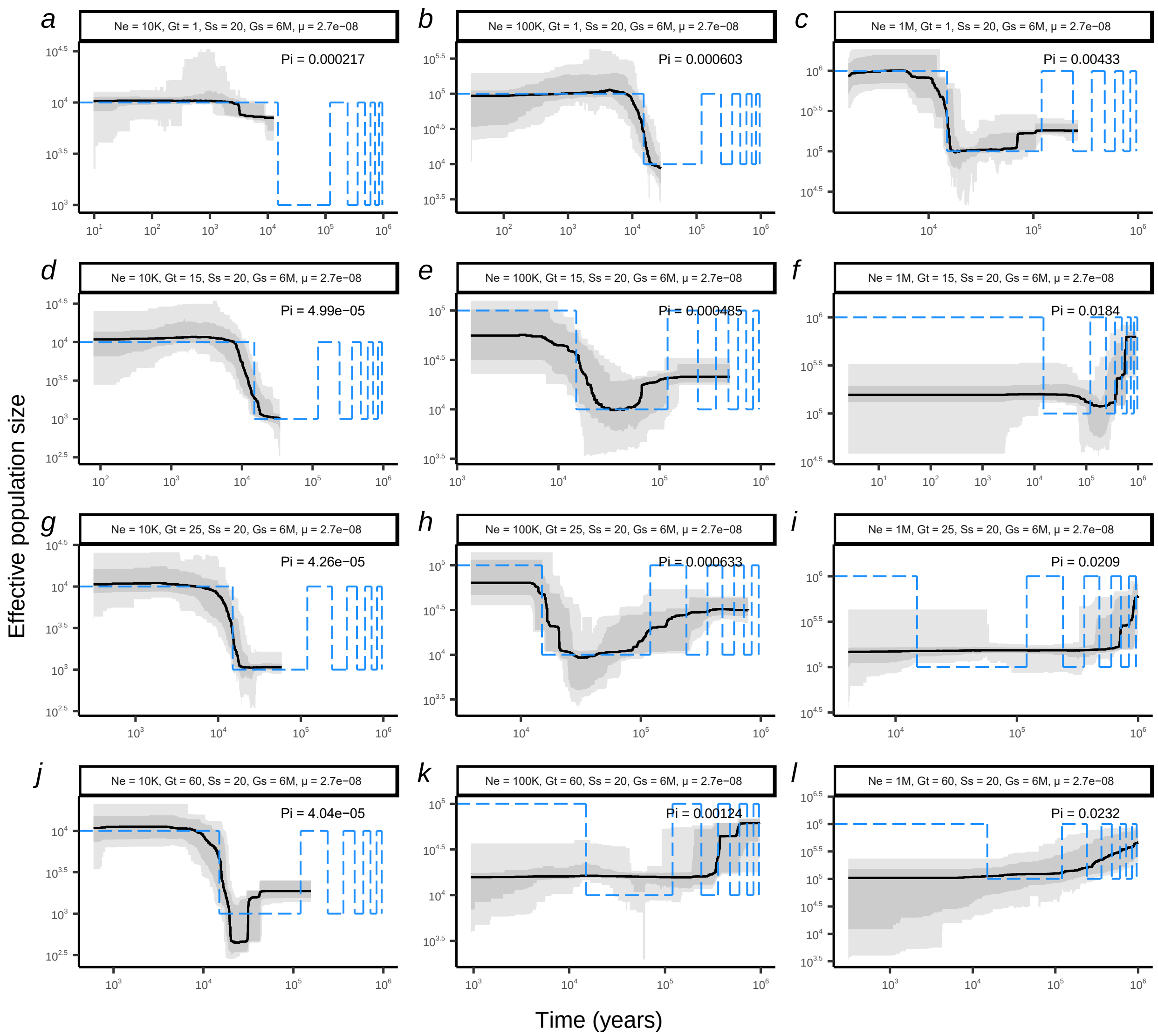

Figure S31. Same as in Fig. S28 but simulations with sample size (Ss) of 20 haploid genomes, with genome size (Gs) of 6 Mbp (60K contigs of length 100 bp) and a mutation rate of 2.7×10^{-8} per site per generation. Source data are provided as a Source Data file-4

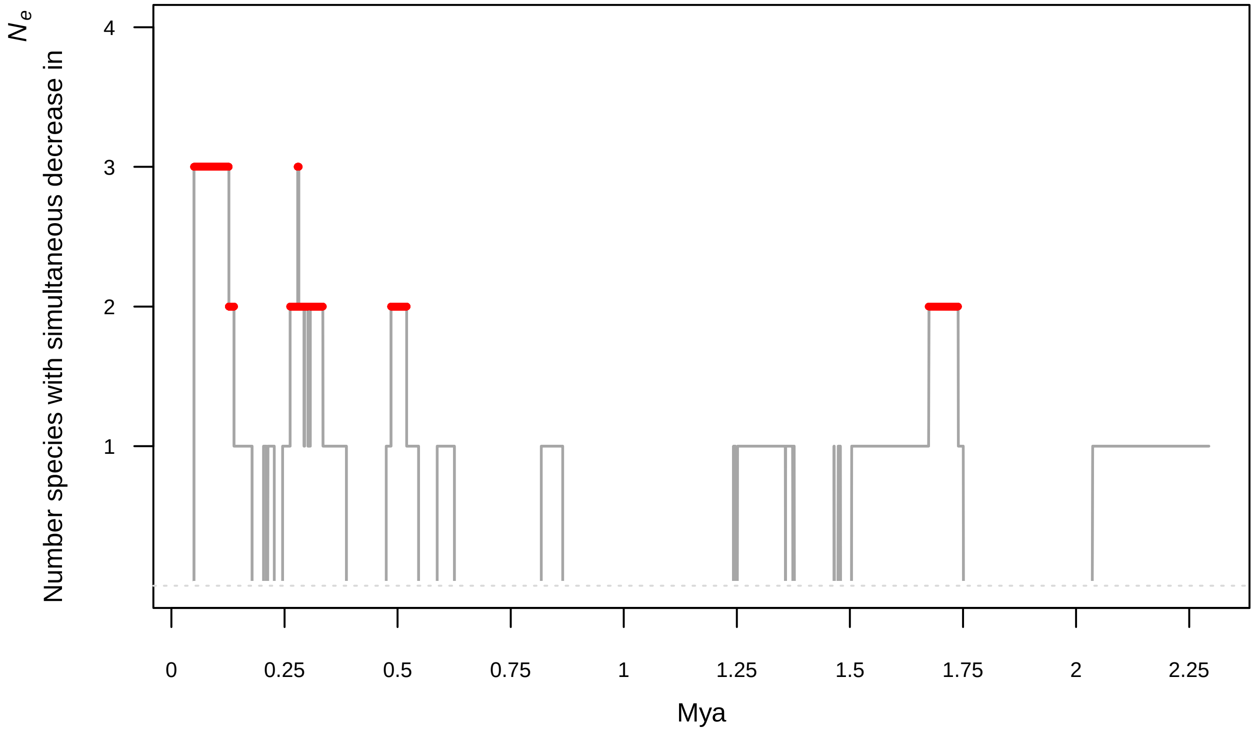

Figure S32. Synchronicity in decreasing phase in N_e between the four species showing the highest correlations in N_e dynamics (*Betula pendula*, *Picea abies*, *Pinus sylvestris* and *Populus nigra*). The solid line represents the number of species experiencing a decrease in N_e at a given time point. The direction of change is given by the average change in N_e across 250 time points using sliding windows see methods section synchronicity analysis. Periods where the synchronicity in decreasing N_e is larger than expected considering the actual change in N_e over time, are highlighted in red. Blue areas along the x axis delineate glacial periods over the last 0.8 Mya. No significant synchronicity in decrease in N_e was observed for *Q. petraea* and *F. sylvatica*. Source data can be found in Tab. S2.

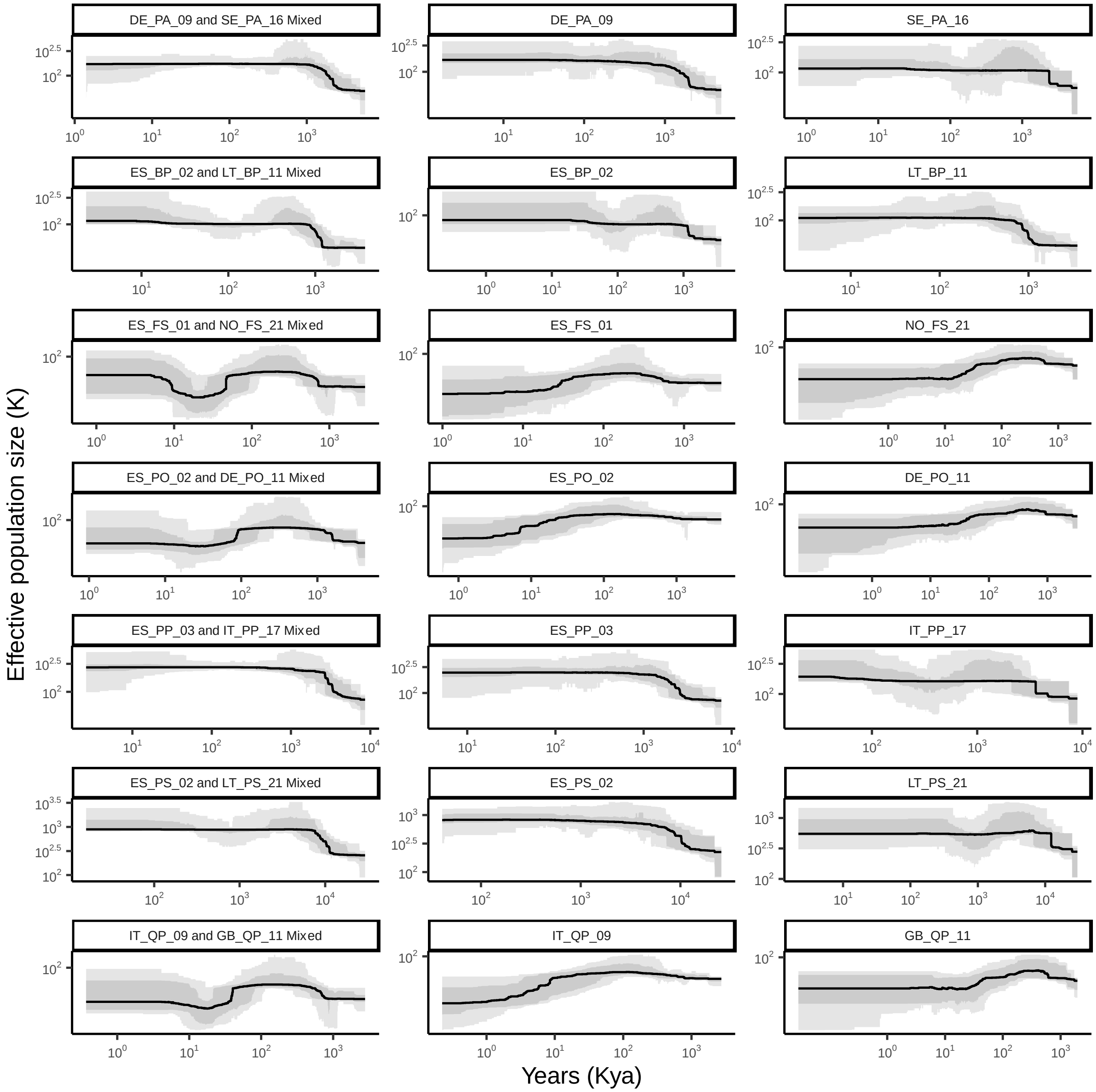

Figure S33. Stairway plot inferences of change in effective population size (N_e) over years (from present to past) and conducted, for each species, on two populations mixed together (leftmost panels), or on each population separately (middle and rightmost panels). From top to bottom, we show the results for *Picea abies*, *Betula pendula*, *Fagus sylvatica*, *Populus nigra*, *Pinus pinaster*, *Pinus sylvestris* and *Quercus petraea*. The populations presented are the same as those used in the manuscript to represent the northern and southern genetic pools (divergence analysis). Source data are provided as a Source Data file-4.

Table S1. Number of populations and individuals per species. The complete list of sampled populations is given in Supplementary Data 1. Populations not included in Opgenoorth et al. (2021)[6] are listed as 'additional populations'.

Species	Populations	Individuals	Additional populations
Betula pendula (BP)	23	497	Belarus (BY_BP_24), Russia (RU_BP_25), Ukraine (UA_BP_23)
Fagus sylvatica (FS)	26	602	Austria (AT_FS_13, AT_FS_14), Slovenia (SI_FS_25, SI_FS_26, SI_FS_27, SI_FS_28)
Populus nigra (PO)	22	467	Great Britain (GB_PO_19), France (FR_PO_21), Morocco (MA_PO_23), Bosnia and Herzegovina (BA_PO_24)
Picea abies (PA)	26	555	Italy (IT_PA_03, IT_PA_04), Norway (NO_PA_22), Poland (PL_PA_25), Romania (RO_PA_24), Russia (RU_OB_01, RU_PA_19, RU_PA_20, RU_PA_26)
Pinus pinaster (PP)	25	472	France (FR_PP_21), Morocco (MA_PP_23, MA_PP_24), Portugal (PT_PP_22), Tunisia (TN_PP_25)
Pinus sylvestris (PS)	23	411	Russia (RU_PS_22, RU_PS_23)
Quercus petraea (QP)	19	403	-

Table S2 Synchronicity in decreasing phase in N_e between the four species showing the highest correlations in N_e dynamics (*Betula pendula*, *Picea abies*, *Pinus sylvestris* and *Populus nigra*). Span (number of consecutive ΔN_e) of synchronous decrease in N_e for a given number of species are compared to the 95th percentiles of the distribution of the maximum spans obtained over 10,000 simulations where observed ΔN_e were directly randomized. Values are bolded and italicized when the duration of a given span is longer than that obtained through simulations, the signal for synchronicity in decreasing N_e is hence considered as being significant. No significant synchronicity in decrease in N_e was observed for *Q. petraea* and *F. sylvatica*.

Two species			Three species		
Years (Kya)		Duration	Years (Kya)		Duration
Start	End		Start	End	
49.8	***138.3***	***661***	49.8	***126.7***	***512***
262.4	***292.9***	***265***	278.9	***281.4***	***23***
294.4	301.8	79	-	-	-
307	***334.6***	***211***	-	-	-
485.3	***520***	***177***	-	-	-
1674	***1738.9***	***131***	-	-	-
95th percentile simulated span		103	95th percentile simulated span		-

Table S3. DNA extraction details for each species.

**Species**	**DNA extraction method**	**Laboratory**
Betula pendula	E.Z.N.A.® SP Plant DNA Kit (Omega Bio-tek)	University of Oulu, Finland
Fagus sylvatica	CTAB	Bavarian Office for Forest Seeding and Planting, ASP, Teisendorf, Germany
Populus nigra	Nucleospin Plant II Mini kit (Macherey-Nagel)	INRAE, BioForA, Orléans, France (except Greek samples extracted by Aristotle University of Tessaloniki, Greece)
Picea abies	DNeasy Plant Mini Kit (QIAGEN)	Uppsala University, Sweden
Pinus pinaster	DNeasy Plant Mini Kit (QIAGEN)	INRAE Biogeco, Bordeaux, France
Pinus sylvestris	DNeasy 96 Plant kit (QIAGEN)	UK Centre for Ecology and Hydrology, UK
Quercus petraea	sbeadex maxi plant kit (LGC Genomics, Berlin, Germany)	Swiss Federal Research Institute WSL, Switzerland

Table S4. Probe design information and statistics.

**Species**	**Reference for probe design**	**Best orthologs (% bp)**	**Other orthologs (% bp)**	**Candidates (% bp)**	**Random (% bp)**	**Criteria for additional targets**
Betula pendula	*Betula pendula* subsp. *pendula* v.1.2 scaffolds, id35079	60.975	-	39.025	-	Genes identified as putative targets of selective sweeps[7]
Fagus sylvatica	*Fagus sylvatica* transcriptome assemblies[8]; *F. sylvatica* candidate gene set[9]	21.644	1.063	7.091	70.202	Genes identified as differentially expressed in drought experiment[8] and during budburst[10]; genes showing adaptive divergence on an elevation gradient[9]
Populus nigra	*Populus trichocarpa* v.3.0 for CDS *Populus nigra* v.1.0 for full genes	65.793	17.659	12.809	3.739	Candidate genes based on annotation and differential expression across populations[11]
Picea abies	*Picea abies* v.1.0	58.365	0.378	4.336	36.921	Orthologous genes between the three conifers; genes identified as putative targets for positive selection[12]
Pinus pinaster	Reference transcriptome at Gymno PLAZA v.1.0	42.276	6.802	10.063	40.859	Orthologous genes between the three conifers; genes with potential roles in adaptation[13–18]; and expressional candidate genes[19]
Pinus sylvestris	Reference transcriptome at Gymno PLAZA v.1.0	48.420	8.846	6.744	35.990	Orthologous genes between the three conifers; genes with potential roles in adaptation[20,21]
Quercus petraea	*Quercus robur* oak haplome v.2.3[22]	41.508	-	28.797	29.695 (14.219 intergenic sequence)	Candidate genes involved in local adaptation for response to water stress[22], and to temperature, precipitation and date of budburst[23]; intergenic sequences corresponding either to SNPs associated

						with local adaptation to temperature, precipitation and date of budburst or to random sequences.

Table S5. Probe design information and statistics.

**Species**	**CDS**	**UTR**	**Unclassified**	**Intergenic**
Betula pendula	1	0	0	0
Fagus sylvatica	1	0	0	0
Populus nigra	1	0	0	0
Picea abies	0.76	0.24	0	0
Pinus pinaster	0.99	0	0.01	0
Pinus sylvestris	0.96	0	0.04	0
Quercus petraea	0.63	0	0	0.37

Table S6. Reference genome used for mapping each species. mt; mitochondrial; cp: chloroplast

**Species**	**Reference genome**	**Reference**
Betula pendula	*Betula pendula* subsp. pendula v.1.2 scaffolds, id35079 (mt: LT855379.1, cp: LT855378.1)	[7]
Fagus sylvatica	*Fagus sylvatica* v.1.3 (cp: NCBI MK598696: mt (*Prunus avium*) NCBI NC_044768.1)	[24]
Populus nigra	*Populus trichocarpa* v.3.1	[25]
Picea abies	*Picea abies* v.1.0	[26]
Pinus pinaster	*Pinus taeda* v.2.01 (mt: NC_039746.1, cp: NC_0214401.1)	[27,28]
Pinus sylvestris	*Pinus taeda* v.2.01 (mt: NC_039746.1, cp: NC_0214401.1)	[27,28]
Quercus petraea	*Quercus robur* v.2.3	[22]

Table S7. Sequencing and mapping statistics. For each species we report the total number of bp sequenced, the number of Illumina reads mapping uniquely in the genome, and the number of bp in the available genome in which at least 50% of the samples of the given species have a minimum coverage of 8x and a minimum genotype quality value of 20.

**Species**	**Gbp sequenced**	**Uniquely mapping reads**	**Available genome (bp)**
Betula pendula	395.41	2,802,450,105	6,041,548
Fagus sylvatica	644.50	2,673,500,437	6,569,688
Populus nigra	507.92	2,435,020,528	6,106,889
Picea abies	706.78	1,517,348,029[a]	4,974,709
Pinus pinaster	1146.77	2,156,038,221	3,068,914
Pinus sylvestris	538.90	1,401,611,778	1,407,443
Quercus petraea	531.30	2,558,016,107	6,323,296

[a] Computed after duplicated reads were removed.

Table S8: Proportion of SNPs in the various categories (SNP set v.5.3.1).

Species	Best-orthologs	Other-orthologs	Candidates	Random	Intergenic
Betula pendula	0.66	0.00	0.26	-	0.08
Fagus sylvatica	0.16	0.01	0.04	0.39	0.40
Picea abies	0.53	0.00	0.05	0.36	0.06
Populus nigra	0.70	0.11	0.08	0.02	0.09
Pinus pinaster	0.17	0.03	0.04	0.17	0.58
Pinus sylvestris	0.13	0.02	0.03	0.11	0.71
Quercus petraea	0.38	0.00	0.12	0.14	0.36

Table S9: Proportion of SNPs in different structural classes (SNP set v.5.3.1).

Species	0-fold	2-3 -fold	4-fold	intergenic	intron	top	up	down
Betula pendula	0.15	0.08	0.10	0.17	0.50	0.00	0.00	0.00
Fagus sylvatica	0.16	0.08	0.10	0.21	0.44	0.00	0.00	0.00
Populus nigra	0.17	0.10	0.11	0.08	0.43	0.00	0.04	0.07
Picea abies	0.20	0.10	0.09	0.41	0.19	0.00	0.00	0.00
Pinus pinaster	0.16	0.08	0.09	0.52	0.16	0.00	0.00	0.00
Pinus sylvestris	0.14	0.06	0.05	0.70	0.05	0.00	0.00	0.00
Quercus petraea	0.10	0.06	0.06	0.39	0.35	0.00	0.02	0.02

Table S10: Number of SNPs is each SNP set and species.

Species	v5.3	v5.3.1	v5.3.2	v6.3.1	v6.3.2
Betula pendula	213250	213250	213250	88741	64697
Fagus sylvatica	223185	166939	166939	54039	40506
Populus nigra	197204	197204	197204	41841	33857
Picea abies	290166	290166	290166	135562	98624
Pinus pinaster	111937	111937	111937	50108	33418
Pinus sylvestris	89903	89903	89903	65205	40650
Quercus petraea	507026	479619	479619	174110	134010

References:

1. Gaunt, T. R., Rodríguez, S. & Day, I. N. M. Cubic exact solutions for the estimation of pairwise haplotype frequencies: implications for linkage disequilibrium analyses and a web tool'CubeX. *BMC Bioinformatics* **8**, 1-9 (2007).
2. Chang, C. C. et al. Second-generation PLINK: rising to the challenge of larger and richer datasets. *GigaScience* **4**, s13742-015 (2015).
3. Alexander, D. H. & Lange, K. Enhancements to the ADMIXTURE algorithm for individual ancestry estimation. *BMC Bioinformatics* **12**, 1-6 (2011).
4. Rellstab, C., Bühler, A., Graf, R., Folly, C. & Gugerli, F. Using joint multivariate analyses of leaf morphology and molecular-genetic markers for taxon identification in three hybridizing European white oak species (Quercus spp.). *Annals of Forest Science* **73**, 669-679 (2016).
5. Caudullo, G., Welk, E. & San-Miguel-Ayanz, J. Chorological data for the main European woody species. *Mendeley Data* **V18**,
6. Opgenoorth, L. et al. The GenTree Platform: growth traits and tree-level environmental data in 12 European forest tree species. *GigaScience* **10**, giab010 (2021).
7. Salojärvi, J. et al. Genome sequencing and population genomic analyses provide insights into the adaptive landscape of silver birch. *Nature Genetics* **49**, 904-912 (2017).
8. Müller, M., Seifert, S., Lübbe, T., Leuschner, C. & Finkeldey, R. De novo transcriptome assembly and analysis of differential gene expression in response to drought in European beech. *PLoS ONE* **12**, e0184167 (2017).
9. Csilléry, K., Rodríguez-Verdugo, A., Rellstab, C. & Guillaume, F. Detecting the genomic signal of polygenic adaptation and the role of epistasis in evolution. *Molecular Ecology* 1-7 (2018).
10. Lesur, I. et al. A unigene set for European beech (*Fagus sylvatica* L.) and its use to decipher the molecular mechanisms involved in dormancy regulation. *Molecular Ecology Resources* **15**, 1192-1204 (2015).
11. Chateigner, A. et al. Gene expression predictions and networks in natural populations supports the omnigenic theory. *BMC Genomics* **21**, 416 (2020).
12. Milesi, P. et al. Assessing the potential for assisted gene flow using past introduction of Norway spruce in southern Sweden: Local adaptation and genetic basis of quantitative traits in trees. *Evolutionary Applications* **12**, 1946-1959 (2019).
13. Eveno, E. et al. Contrasting patterns of selection at *Pinus pinaster* Ait. drought stress candidate genes as revealed by genetic differentiation analyses. *Molecular Biology and Evolution* **25**, 417-437 (2008).
14. Grivet, D. et al. Molecular footprints of local adaptation in two Mediterranean conifers. *Molecular Biology and Evolution* **28**, 101-116 (2011).
15. Grivet, D. et al. High rate of adaptive evolution in two widespread European pines. *Molecular Ecology* **26**, 6857-6870 (2017).
16. Jaramillo-Correa, J.-P. et al. Molecular proxies for climate maladaptation in a long-lived tree (*Pinus pinaster* Aiton, Pinaceae). *Genetics* **199**, 793-807 (2015).
17. Cabezas, J. A. et al. Nucleotide polymorphisms in a pine ortholog of the Arabidopsis degrading enzyme cellulase KORRIGAN are associated with early growth performance in *Pinus pinaster*. *Tree Physiology* **35**, 1000-1006 (2015).
18. Plomion, C. et al. High-density SNP assay development for genetic analysis in maritime pine (*Pinus pinaster*). *Molecular Ecology Resources* **16**, 574-587 (2016).

19. Cañas, R. A., Feito, I., Ávila, C., Majada, J. & Cánovas, F. M. Transcriptome-wide analysis supports environmental adaptations of two *Pinus pinaster* populations from contrasting habitats. *BMC Genomics* **16**, 1-17 (2015).
20. Wachowiak, W., Trivedi, U., Perry, A. & Cavers, S. Comparative transcriptomics of a complex of four European pine species. *BMC Genomics* **16**, 234 (2015).
21. Wachowiak, W., Salmela, M. J., Ennos, R. A., Iason, G. & Cavers, S. High genetic diversity at the extreme range edge: Nucleotide variation at nuclear loci in Scots pine (*Pinus sylvestris* L.) in Scotland. *Heredity* **106**, 775-787 (2011).
22. Plomion, C. et al. Oak genome reveals facets of long lifespan. *Nature Plants* **4**, 440-452 (2018).
23. Rellstab, C. et al. Signatures of local adaptation in candidate genes of oaks (Quercus spp.) with respect to present and future climatic conditions. *Molecular Ecology* **25**, 5907-5924 (2016).
24. Mishra, B. et al. A reference genome of the European beech (*Fagus sylvatica* L.). *GigaScience* **7**, giy063 (2018).
25. Tuskan, G. A. et al. The genome of black cottonwood, *Populus trichocarpa* (Torr. & Gray). *Science* **313**, 1596-1604 (2006).
26. Nystedt, B. et al. The Norway spruce genome sequence and conifer genome evolution. *Nature* **497**, 579-584 (2013).
27. Wegrzyn, J. L. et al. Unique features of the loblolly pine (*Pinus taeda* L.) megagenome revealed through sequence annotation. *Genetics* **196**, 891-909 (2014).
28. Neale, D. B. et al. Decoding the massive genome of loblolly pine using haploid DNA and novel assembly strategies. *Genome Biology* **15**, R59 (2014).
