# Supplementary material for: Resilience of genetic diversity in forest trees over the Quaternary
Source: Nat Commun. 2024 Oct 14;15:8538. doi: 10.1038/s41467-024-52612-y (PMC11473659; doi:10.1038/s41467-024-52612-y)
Supplement: Supplementary file 3 — Reporting Summary [file 41467_2024_52612_MOESM3_ESM.pdf]

nature portfolio

Corresponding author(s): Tanja Pyhäjärvi, Pascal Milesi, Martin Lascoux

Last updated by author(s): Aug 24, 2024

Reporting Summary

Nature Portfolio wishes to improve the reproducibility of the work that we publish. This form provides structure for consistency and transparency in reporting. For further information on Nature Portfolio policies, see our Editorial Policies and the Editorial Policy Checklist.

Statistics

For all statistical analyses, confirm that the following items are present in the figure legend, table legend, main text, or Methods section.

n/a	Confirmed	
☐	☒	The exact sample size (n) for each experimental group/condition, given as a discrete number and unit of measurement
☒	☐	A statement on whether measurements were taken from distinct samples or whether the same sample was measured repeatedly
☐	☒	The statistical test(s) used AND whether they are one- or two-sided<br>*Only common tests should be described solely by name; describe more complex techniques in the Methods section.*
☐	☒	A description of all covariates tested
☐	☒	A description of any assumptions or corrections, such as tests of normality and adjustment for multiple comparisons
☐	☒	A full description of the statistical parameters including central tendency (e.g. means) or other basic estimates (e.g. regression coefficient) AND variation (e.g. standard deviation) or associated estimates of uncertainty (e.g. confidence intervals)
☐	☒	For null hypothesis testing, the test statistic (e.g. F, t, r) with confidence intervals, effect sizes, degrees of freedom and P value noted<br>*Give P values as exact values whenever suitable.*
☒	☐	For Bayesian analysis, information on the choice of priors and Markov chain Monte Carlo settings
☐	☒	For hierarchical and complex designs, identification of the appropriate level for tests and full reporting of outcomes
☒	☐	Estimates of effect sizes (e.g. Cohen's d, Pearson's r), indicating how they were calculated

Our web collection on statistics for biologists contains articles on many of the points above.

Software and code

Policy information about availability of computer code

Data collection	no software was used for data collection
Data analysis	BLAST v.2.5.0+, Orthofinder v.1.1.4, SSAHA algorithm, Illumina bcl2fastq v.2.20, ERNE v.1.4.6, Cutadapt, BWA mem v.0.7.17, samtools v.1.7, Picard, GATK v.4.0.10.0, ADMIXTURE v.1.3, HDplot, NewAnnotateRef.py, ANNOVAR, raster R package v.3.1.5, StAMPP R package v.1.6.3, poppr R package v.2.9.3, vcftools (v.0.1.13), geosphere R package v.1.5-10, EIGENSOFT v.7.2.0, fastsimcoal2, Stairway Plot 2 v.2.1.1,

For manuscripts utilizing custom algorithms or software that are central to the research but not yet described in published literature, software must be made available to editors and reviewers. We strongly encourage code deposition in a community repository (e.g. GitHub). See the Nature Portfolio guidelines for submitting code & software for further information.

Data

Policy information about availability of data

All manuscripts must include a data availability statement. This statement should provide the following information, where applicable:

- Accession codes, unique identifiers, or web links for publicly available datasets
- A description of any restrictions on data availability
- For clinical datasets or third party data, please ensure that the statement adheres to our policy

The short read data generated in this study have been deposited to NCBI BioProjects under accession codes PRJNA602465[https://www.ncbi.nlm.nih.gov/bioproject/?term=PRJNA602465], PRJNA602466[https://www.ncbi.nlm.nih.gov/bioproject/?term=PRJNA602466], PRJNA602467[https://www.ncbi.nlm.nih.gov/

bioproject/?term=PRJNA602467], PRJNA602468[https://www.ncbi.nlm.nih.gov/bioproject/?term=PRJNA602468], PRJNA602470[https://www.ncbi.nlm.nih.gov/bioproject/?term=PRJNA602470], PRJNA602471[https://www.ncbi.nlm.nih.gov/bioproject/?term=PRJNA602471], PRJNA602473[https://www.ncbi.nlm.nih.gov/bioproject/?term=PRJNA602473]. The vcf, ped and map -files generated in this study are available in Data INRAE at https://doi.org/10.57745/DV2X0M. Source data are provided as a Source Data file.

Research involving human participants, their data, or biological material

Policy information about studies with human participants or human data. See also policy information about sex, gender (identity/presentation), and sexual orientation and race, ethnicity and racism.

Reporting on sex and gender	*Use the terms sex (biological attribute) and gender (shaped by social and cultural circumstances) carefully in order to avoid confusing both terms. Indicate if findings apply to only one sex or gender; describe whether sex and gender were considered in study design; whether sex and/or gender was determined based on self-reporting or assigned and methods used. Provide in the source data disaggregated sex and gender data, where this information has been collected, and if consent has been obtained for sharing of individual-level data; provide overall numbers in this Reporting Summary. Please state if this information has not been collected. Report sex- and gender-based analyses where performed, justify reasons for lack of sex- and gender-based analysis.*
Reporting on race, ethnicity, or other socially relevant groupings	*Please specify the socially constructed or socially relevant categorization variable(s) used in your manuscript and explain why they were used. Please note that such variables should not be used as proxies for other socially constructed/relevant variables (for example, race or ethnicity should not be used as a proxy for socioeconomic status). Provide clear definitions of the relevant terms used, how they were provided (by the participants/respondents, the researchers, or third parties), and the method(s) used to classify people into the different categories (e.g. self-report, census or administrative data, social media data, etc.) Please provide details about how you controlled for confounding variables in your analyses.*
Population characteristics	*Describe the covariate-relevant population characteristics of the human research participants (e.g. age, genotypic information, past and current diagnosis and treatment categories). If you filled out the behavioural & social sciences study design questions and have nothing to add here, write "See above."*
Recruitment	*Describe how participants were recruited. Outline any potential self-selection bias or other biases that may be present and how these are likely to impact results.*
Ethics oversight	*Identify the organization(s) that approved the study protocol.*

Note that full information on the approval of the study protocol must also be provided in the manuscript.

Field-specific reporting

Please select the one below that is the best fit for your research. If you are not sure, read the appropriate sections before making your selection.

☐ Life sciences ☐ Behavioural & social sciences ☒ Ecological, evolutionary & environmental sciences

For a reference copy of the document with all sections, see nature.com/documents/nr-reporting-summary-flat.pdf

Ecological, evolutionary & environmental sciences study design

All studies must disclose on these points even when the disclosure is negative.

Study description	The study is a comparative genomics analysis of the genetic diversity and demography history of seven forest trees species. We perform comparative population genomic analyses and demographic inferences for seven widely distributed and ecologically contrasting European forest tree species based on concerted sampling of 164 populations across their natural ranges. We conducted targeted nuclear DNA sequencing (~10,000 species-specific probes that covered ~3 Mbp of largely orthologous sequences) on a total of 3,407 adult trees collected from 19 to 26 locations per species (~25 individuals each) across large parts of their natural ranges. We conducted a comprehensive survey of the distribution of current genetic diversity in all seven species and used coalescent approaches to reconstruct changes in Ne over multiple glacial cycles and test for synchronous changes across species.
Research sample	Sampling location are reported in the supporting information Table S1.
Sampling strategy	We used samples and sampling described in detail in Opgenoorth et al. 2021 (https://academic.oup.com/gigascience/article/10/3/giab010/6177710). We sampled a minimum of 20 individuals per location. Note that in the coalescent framework , adding more samples beyond 10 does not add much more information about the population history (https://doi.org/10.1093/molbev/msj079). However, adding more loci improves the accuracy of our population genetic parameter estimation as it allows independent estimation of parameters across the genome, because loci's coalescent histories are separated by recombination.
Data collection	Tissue collection is described in detail in Opgenoorth et al. 2021 (https://academic.oup.com/gigascience/article/10/3/giab010/6177710). DNA extractions were carried out in University of Oulu, Finland, Bavarian Office for Forest Seeding and Planting, ASP, Teisendorf, Germany, INRAE, BioForA, Orléans, France (except Greek samples extracted by Aristotle University of Tessaloniki, Greece), Uppsala University, Sweden, NRAE Biogeco, Bordeaux, France, UK Centre for Ecology and Hydrology, UK, and Swiss Federal Research Institute WSL, Switzerland. DNA sequencing was coordinated and conducted by 21IGA Technology Services S.r.l., 33100 Udine, Italy. To estimate the quality of genomic DNA, we quantified random samples from each 96-well plate using a Qubit 2.0

	Fluorometer (Invitrogen, Carlsbad, CA, USA) and a NanoDrop 1000 spectrophotometer (Thermo Fisher Scientific, Waltham, MA, USA). We quantified all 4,754 samples using the GloMax Explorer System (Promega Corporation, Madison, WI, USA) and prepared libraries for target enrichment using the SeqCap EZ – HyperPlus kit (Roche Sequencing Solutions, Pleasanton, CA, USA) with 100 ng/µl of input DNA, following the manufacturer's instructions. For P. abies we conducted a second round of library preparation using 200 ng/µL of input DNA. We evaluated library size using the Bioanalyzer High Sensitivity DNA assay (Agilent Technologies, Santa Clara, CA, USA) and quantified libraries using a Qubit 2.0 Fluorometer. We sequenced the libraries on a HiSeq 2500 (125 cycles per read) for P. abies and B. pendula and on a NovaSeq 6000 (Illumina, San Diego, CA, USA; 150 cycles per read) for the remaining species, in both cases working in paired-end mode. We used Illumina bcl2fastq v.2.20 for base calling and demultiplexing, and we used ERNE v.1.4.6 and Cutadapt for quality and adapter trimming, both with default parameters.
Timing and spatial scale	Empirical data has been collected 2016-2020 across Europe, Russia and North Africa
Data exclusions	Some samples were excluded during early filtering step based on genotype information. The rationale is fully developed in the method section of the manuscript
Reproducibility	This study focuses on genetic variation found in natural populations by collecting more than 400 samples per species across a large fraction of their distribution. The natural evolution of these species cannot be reproduced. Most of our analyses were repeated and cross-validated by combining several methods. Code to repeat our analyses are made available.
Randomization	Not part of the methodology we used for obtaining the primary data.
Blinding	Investigators were not blinded during data collection and analysis. The methodology we used was not sensitive to human a priori information or knowledge about the samples. For example, there is no human impact on measures of population structure or genetic diversity. In addition, it would have been impossible to conduct sampling and analysis blinded. Rather, the investigators needed to know the origin of samples to e.g., identify and remove interspecific hybrids from the data.

Did the study involve field work? ☐ Yes ☒ No

Reporting for specific materials, systems and methods

We require information from authors about some types of materials, experimental systems and methods used in many studies. Here, indicate whether each material, system or method listed is relevant to your study. If you are not sure if a list item applies to your research, read the appropriate section before selecting a response.

Materials & experimental systems

n/a	Involved in the study	
☒	☐	Antibodies
☒	☐	Eukaryotic cell lines
☒	☐	Palaeontology and archaeology
☒	☐	Animals and other organisms
☒	☐	Clinical data
☒	☐	Dual use research of concern
☐	☒	Plants

Methods

n/a	Involved in the study	
☒	☐	ChIP-seq
☒	☐	Flow cytometry
☒	☐	MRI-based neuroimaging

Dual use research of concern

Policy information about dual use research of concern

Hazards

Could the accidental, deliberate or reckless misuse of agents or technologies generated in the work, or the application of information presented in the manuscript, pose a threat to:

No	Yes	
☒	☐	Public health
☒	☐	National security
☒	☐	Crops and/or livestock
☒	☐	Ecosystems
☒	☐	Any other significant area

Experiments of concern

Does the work involve any of these experiments of concern:

No	Yes	
☒	☐	Demonstrate how to render a vaccine ineffective
☒	☐	Confer resistance to therapeutically useful antibiotics or antiviral agents
☒	☐	Enhance the virulence of a pathogen or render a nonpathogen virulent
☒	☐	Increase transmissibility of a pathogen
☒	☐	Alter the host range of a pathogen
☒	☐	Enable evasion of diagnostic/detection modalities
☒	☐	Enable the weaponization of a biological agent or toxin
☒	☐	Any other potentially harmful combination of experiments and agents

Plants

Seed stocks	All the material was collected from the field. Details are described in Opgenoorth et al. 2021 (https://academic.oup.com/gigascience/article/10/3/giab010/6177710#supplementary-data).
Novel plant genotypes	We did not create any novel plant genotypes
Authentication	We did not create any novel plant genotypes
