# Supplementary material for: Resilience of genetic diversity in forest trees over the Quaternary
Source: Nat Commun. 2024 Oct 14;15:8538. doi: 10.1038/s41467-024-52612-y (PMC11473659; doi:10.1038/s41467-024-52612-y)
Supplement: Supplementary file 4 — Description of Additional Supplementary Files [file 41467_2024_52612_MOESM4_ESM.pdf]

Description of Additional Supplementary Files

File Name: Supplementary Data 1

Description: Sampling location IDs, species, coordinates (WGS84) and number of individuals in vcf v.5.3.1.

File Name: Supplementary Data 2

Description: Coordinates, expected heterozygosity at all, silent, 4-fold and 0-fold degenerate sites, and population-specific FST per population and species.

File Name: Supplementary Data 3

Description: Genetic distance and geographic distance (as pairwise FST) among all pairs of populations.

File Name: Supplementary Data 4

Description: AIC, delta AIC and wi computed for each species, sampling design and model.

File Name: Supplementary Data 5

Description: Confidence intervals (95%) of the parameter obtained with the panmictic models (2-epoch and 3-epoch, with one and two demographic changes, respectively) across all species and the two sampling designs (AllSamples = all samples included, OnePerPop = one haplotype per location included). The median and the 2.5 and 97.5 percentiles are provided for each parameter. NANC = population effective size of the ancestral population; NCUR = population effective size of the current population; TLATE = time in generations of the demographic event in the 2-epoch model and the most ancient demographic event in the 3-epoch model; TEARLY = time in generations of the most recent demographic event in the 3-epoch model; TLATE_year = same as TLATE but expressed in years; TEARLY_year = same as TLATE but expressed in year; Ratio NCUR/NANC = ratio of NCUR and NANC.

File Name: Supplementary Data 6

Description: Single-point estimates for several parameters obtained from simulations under seven models (SNM, 2-epoch, 3-epoch, div-e1-iso, div-e2-iso, div-e1-mig, div-e2-mig) across four sampling designs (AllSamples, OnePerPop, PairPop, SinglePop) with fastsimcoal2. These estimates were taken from the best run out of 100 independent runs (see Materials and Methods).

File Name: Supplementary Data 7

Description: All results obtained with Stairway Plot 2 concatenated into a single file with columns ‘species’ and ‘population’ specific to each individual analysis. Species-wide analyses were run using two sampling designs, AllSamples and OnePerPop, and are identified as such in the ‘population’ column. For population-wide analyses the population ID is given in the ‘population’ column.

File Name: Supplementary Data 8

Description: Gene Ontology (GO) and Kyoto Encyclopedia of Genes and Genomes (KEGG) terms for selected genes.

File Name: Supplementary Data 9

Description: The set of 811 orthogroups that included at least one gene for at least six of the seven tree species.

File Name: Supplementary Data 10

Description: Other ‘orthogroups’ including genes that were found only in a reduced set of species (e.g. 59 additional genes across the three conifer species).
